# Supplementary material for: Cancer cells adapt FAM134B/BiP mediated ER-phagy to survive hypoxic stress
Source: Cell Death Dis. 2022 Apr 18;13(4):357. doi: 10.1038/s41419-022-04813-w (PMC9016075; doi:10.1038/s41419-022-04813-w)
Supplement: Supplementary file 1 — Supplemental Figures, Legends and Table [file 41419_2022_4813_MOESM1_ESM.pdf]

Supplementary Fig. 1

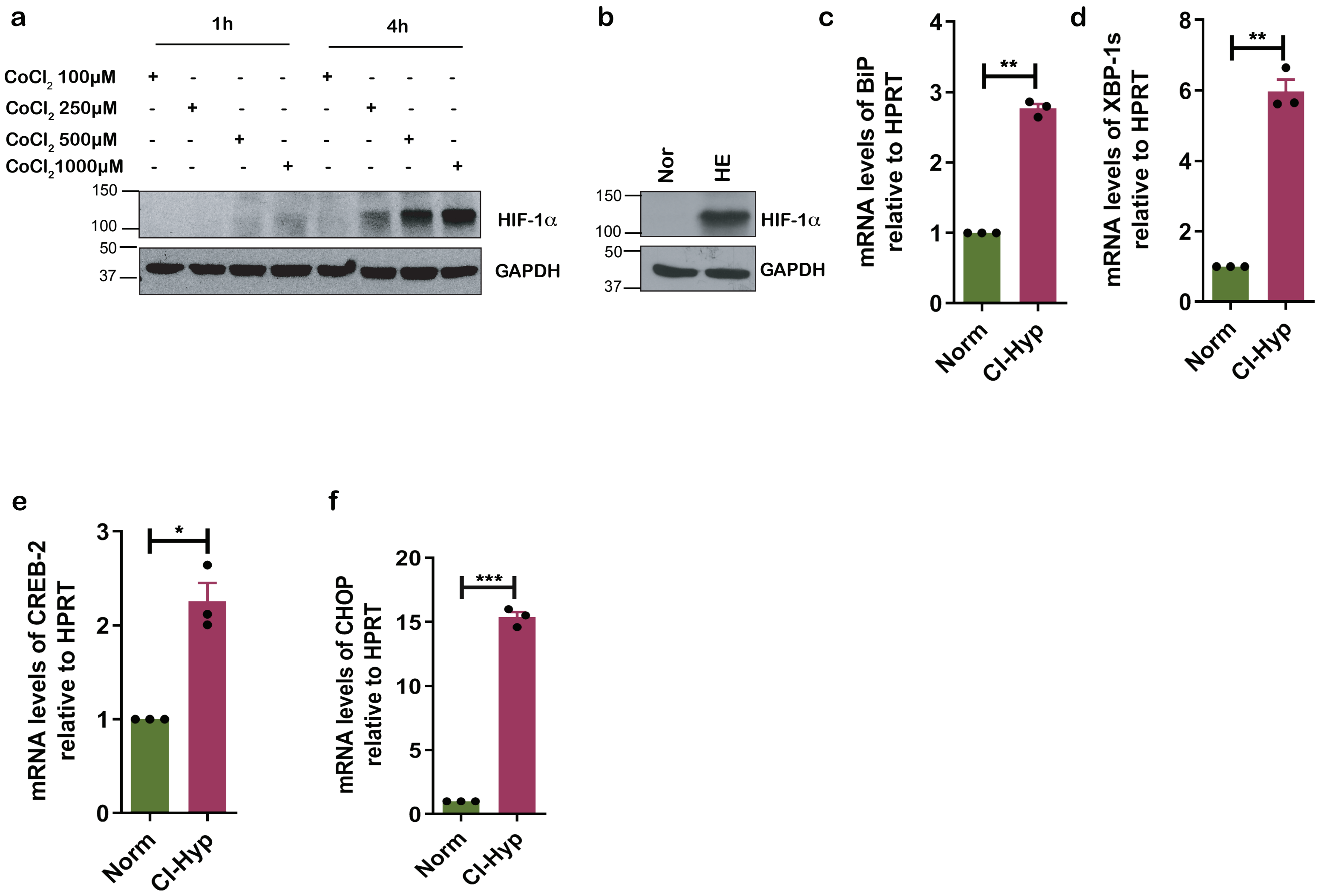

Supplementary Fig. 2

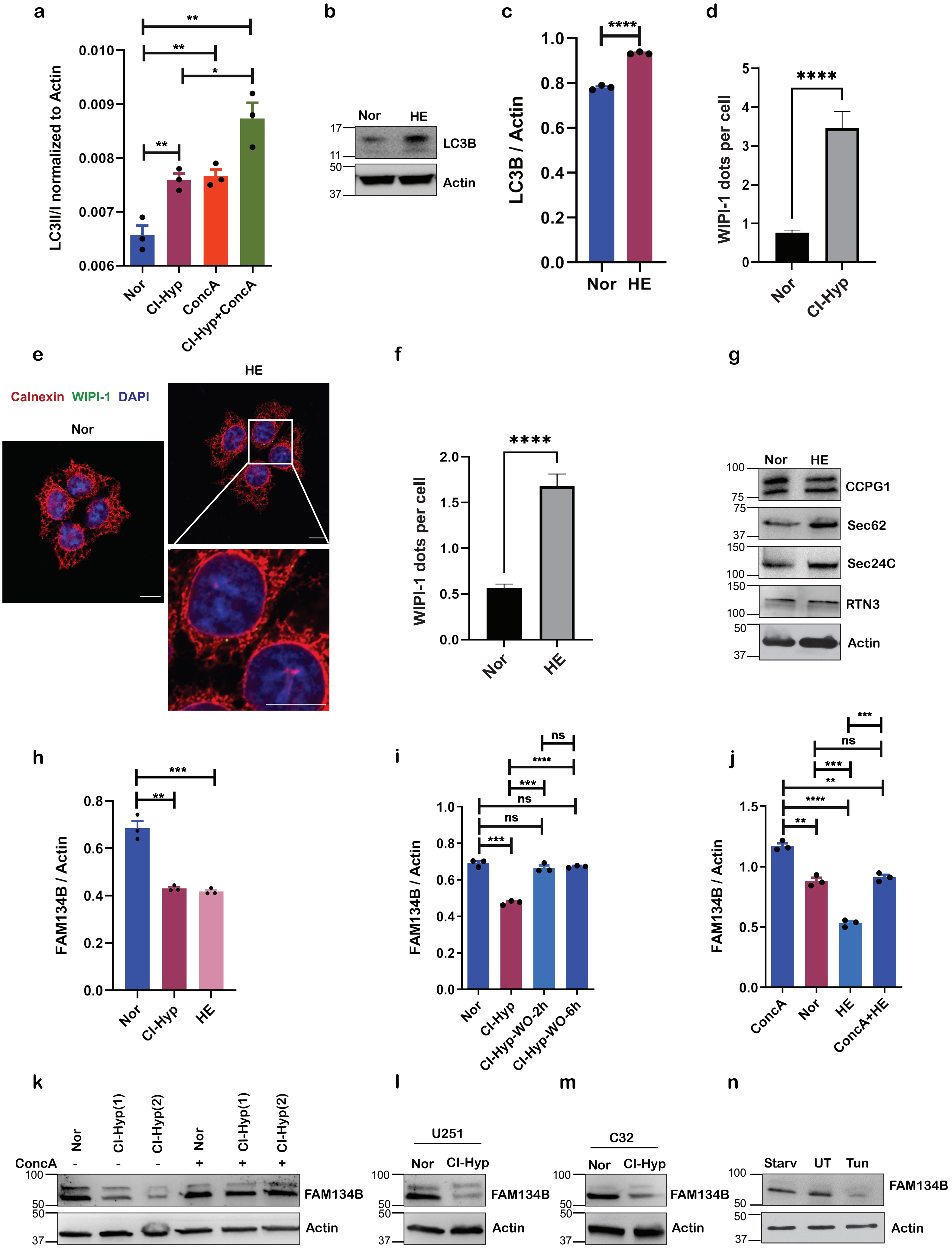

Supplementary Fig. 3

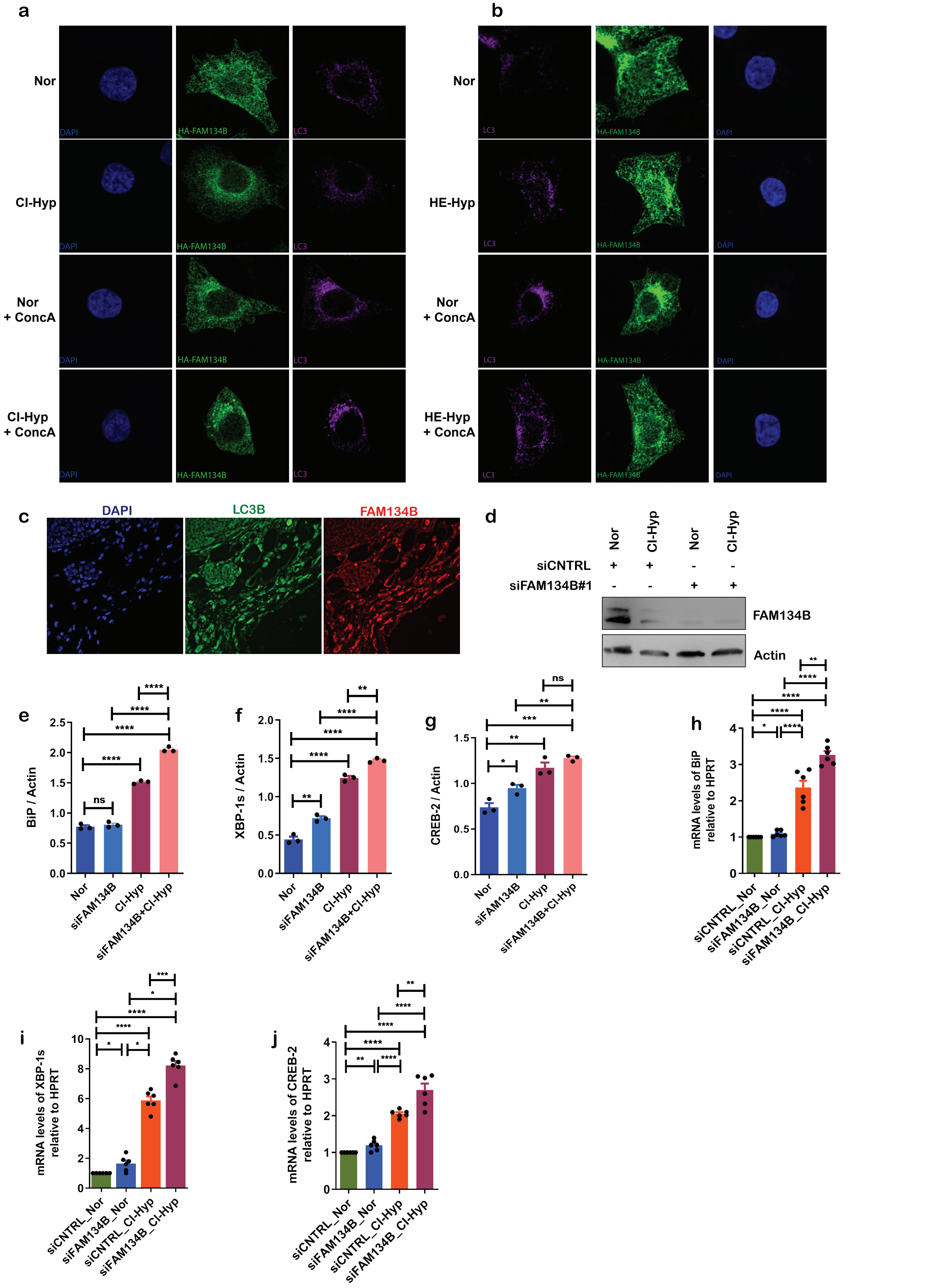

Supplementary Fig. 4

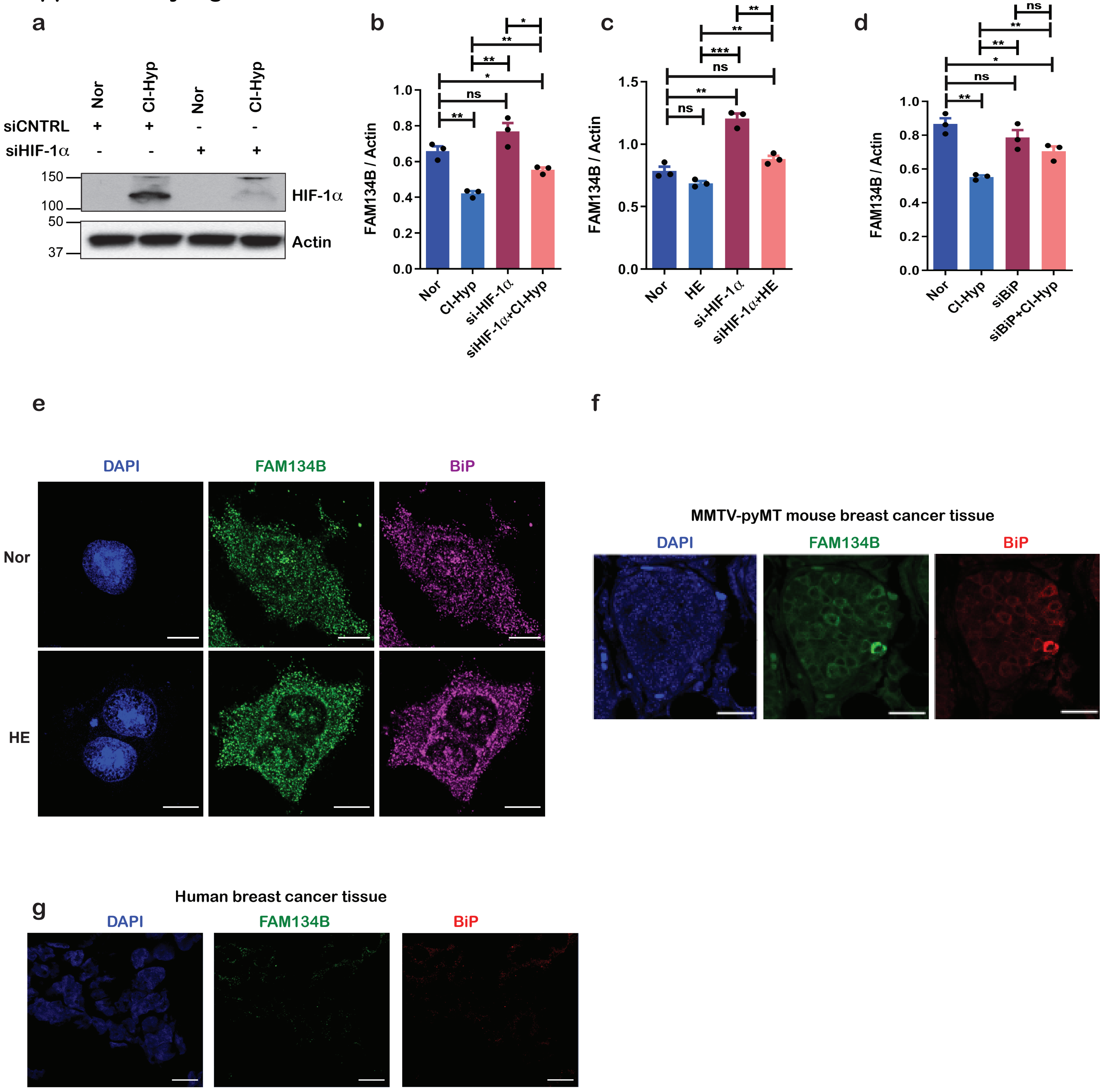

Supplementary Fig. 5

Supplementary Fig. 5

a

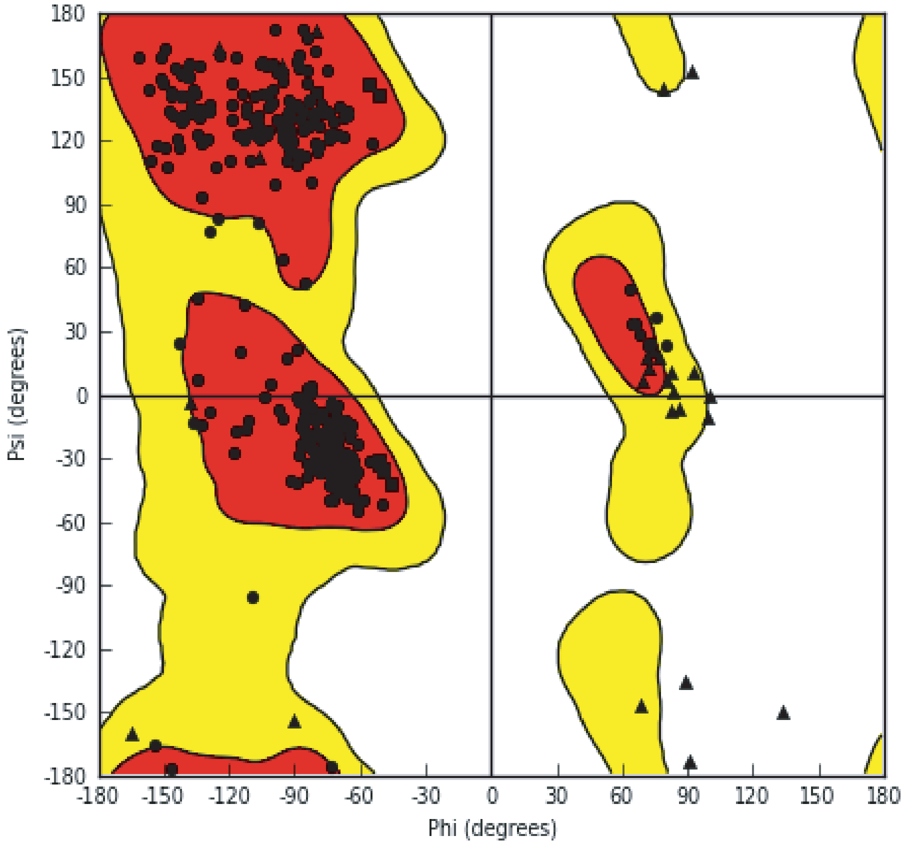

b

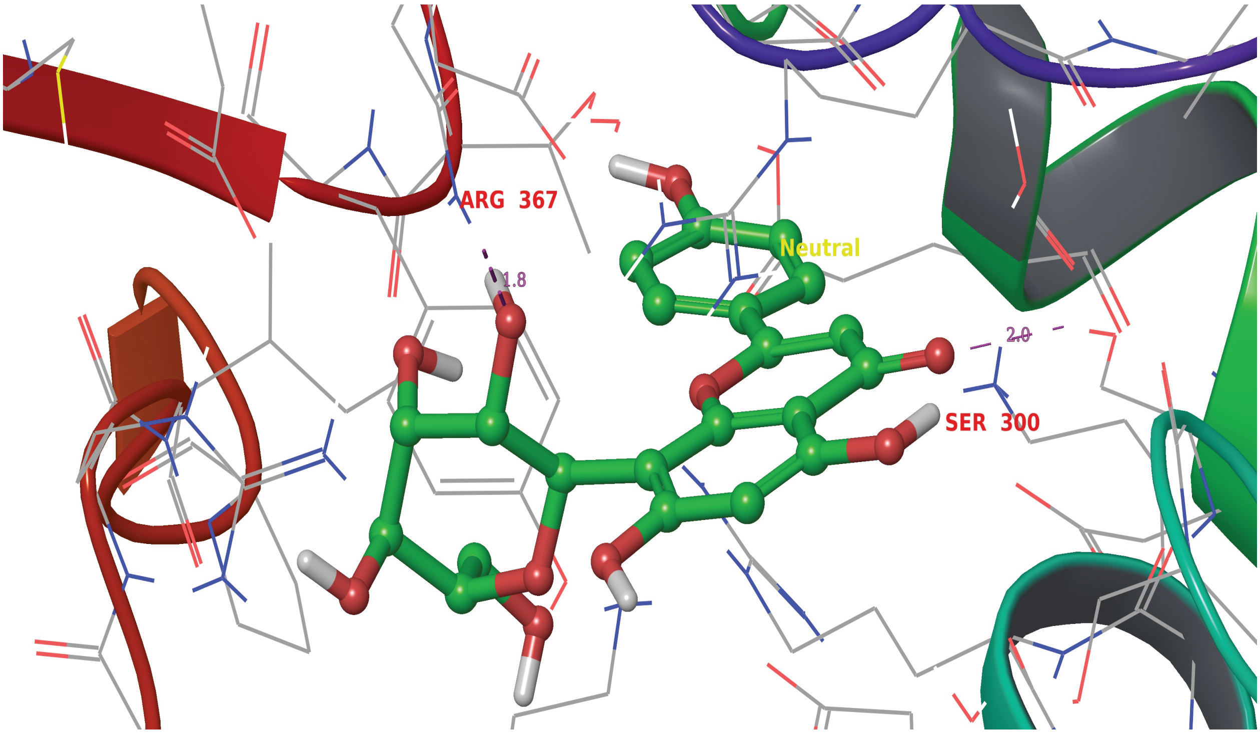

Supplementary Fig. 6

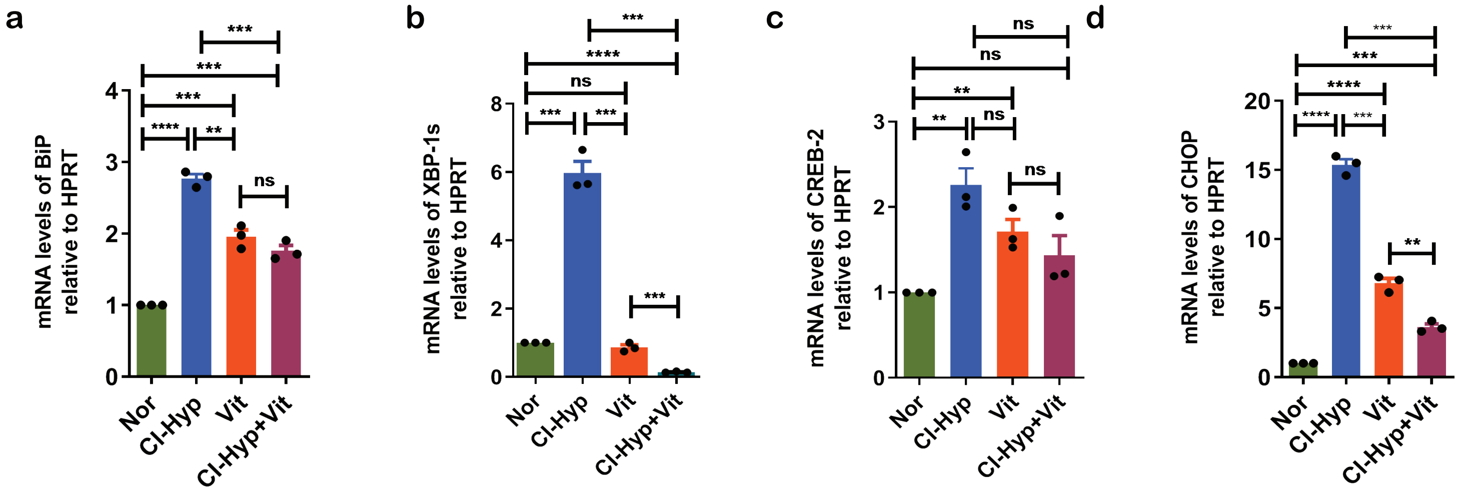

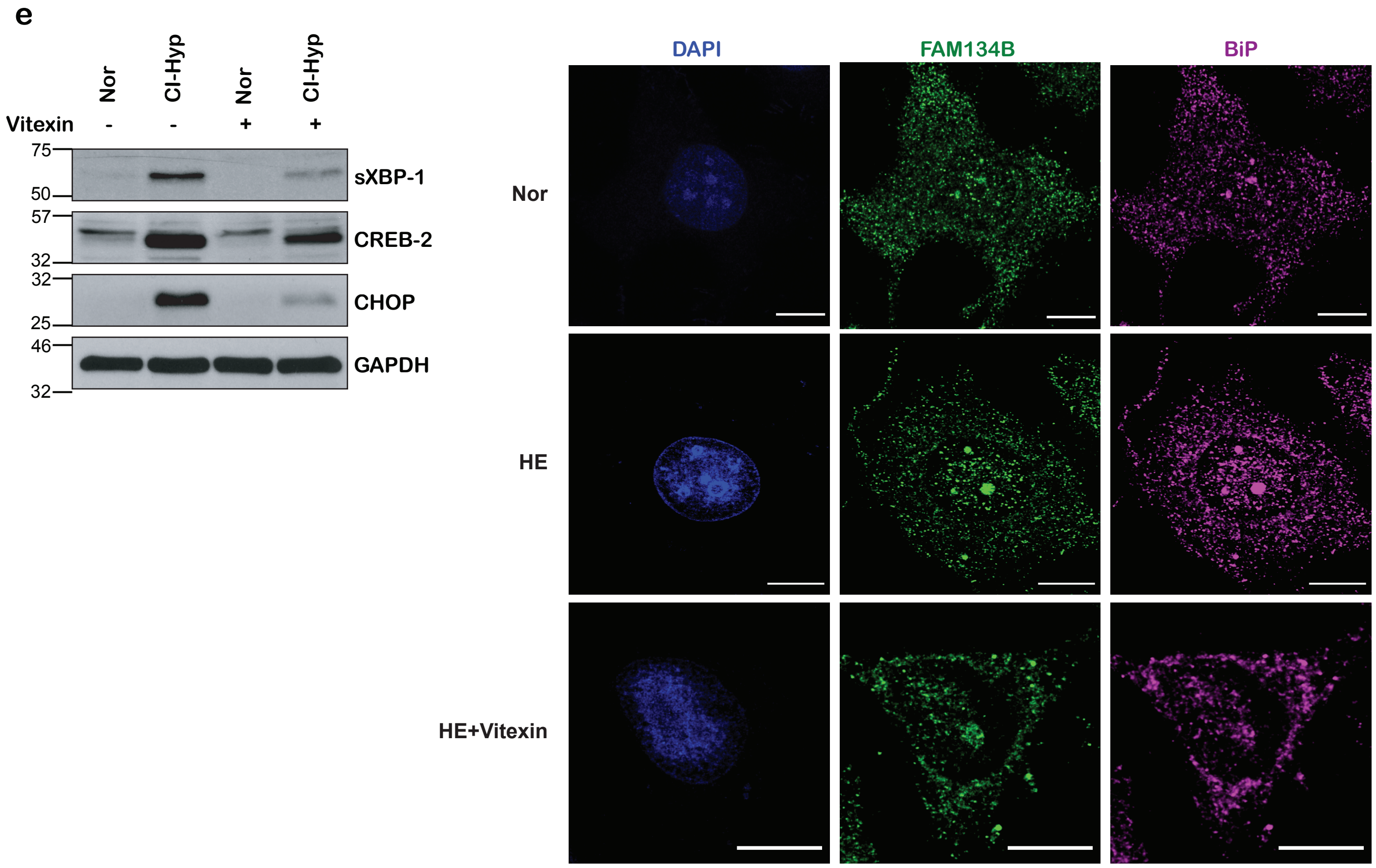

Supplementary Figure Legends

Supplementary Fig. 1: (a) Immunoblot showing concentration dependent increase in HIF-1a expression in MCF-7 cells after 1h and 4h of CoCl2 treatment *(n=3)*. **(b)** Induction of HIF-1a in MCF-7 cells in HE (1% O_2) (*n=3*). **(c-f)** Relative mRNA expression of UPR markers **(c)** BiP, **(d)** XBP-1s, **(e)** ATF4 & **(f)** CHOP respectively *(n=3)*.

Supplementary Fig. 2: (a) Densitometric quantification of LC3I to LC3II conversion upon concanamycin A treatment during CI-hypoxia in comparison to control *(n=3)*. **(b)** Immunoblot expression of LC3B upon HE (1% O_2) in MCF-7 cells *(n=3)*. **(c)** Densitometric quantification of LC3B upon HE (1% O_2) in MCF-7 cells *(n=3)*. **(d)** Graph depicts the number of WIPI-1 dots colocalizing with ER (Fig. 2b) per cell in normoxic cells and CI-hypoxic cells. **(e)** Confocal image of MCF-7 cells cultured in HE stained for calnexin (ER) and WIPI (autophagosomes). **(f)** Graph depicts the number of WIPI dots colocalizing with ER per cell in normoxic cells and cells in HE. **(g)** Immunoblot of ER-phagy receptors SEC62, CCPG1, SEC24C and RTN3 during hypoxia *(n=3)*. **(h)** Densitometric quantification of FAM134B during CI-hypoxia and HE (1% O_2) compared to normoxia **(Fig. 2g)** *(n=5)*. **(i)** Densitometric analysis of FAM134B expression in the presence of CoCl2 and after CoCl2 wash out *(n=3)*. **(j)** Densitometric quantification of FAM134B during HE (1% O2) in the presence and absence of concanamycin A *(n=4)*. **(k)** Western blot of FAM134B in MCF-7 cells treated with concanamycin A during CI-hypoxia *(n=3)*. **(l)** Immunoblot of FAM134B degradation during CI-hypoxia in U251 and **(m)** C32 cells *(n=3)*. **(n) FAM134B expression in MCF-7 cells subjected to starvation (Starv) or drug induced ER-stress using tunicamycin (Tun).**

Supplementary Fig. 3: (a) Individual channels of immunofluorescent staining for HA-FAM134B and LC3B in MCF-7 cells subjected to CI-hypoxia or (**b**) HE in the presence or absence of concanamycin A **(c) Individual channels of** MMTV-pyMT mouse breast cancer tissue sections stained for FAM134B (red), LC3B (green) and nucleus (blue). **(d)** Immunoblot of FAM134B in MCF-7 cells transfected with *FAM134B* specific siRNA *(n=3)*. **(e-g)** Densitometric quantification of **(e)** BiP, **(f)** XBP-1s and **(g)** ATF4 upon FAM134B depletion during CI-hypoxia *(n=3)*. **(h-j)** Relative mRNA expression of UPR markers **(h)** BiP, **(i)** XBP-1s, **(j)** ATF4 *(n=3)* in MCF-7 cells depleted or FAM134B using siRNA and subjected to CI-hypoxia.

Supplementary Fig. 4: (a) Immunoblot of HIF-1a in MCF-7 cells transfected with *HIF-1a* specific siRNA *(n=3)*. **(b)** Densitometric quantification of FAM134B expression in HIF-1a siRNA transfected cells during CI-hypoxia and **(c)** HE (1% O2) *(n=3)*. **(d)** Densitometric quantification of FAM134B during CI-hypoxia upon silencing BiP *(n=3)*. **(e)** Individual channels - confocal immunofluorescence image of MCF-7 cells cultured in HE stained for FAM134B (green), BiP (purple) and nucleus – DAPI (blue). **(f)** Individual channels from confocal image of MMTV-pyMT breast cancer tissue section stained for FAM134B and BiP **(Fig. 3j)**; scale bar = 20um. **(g)** Individual channels from confocal image of human breast cancer tissue section stained for FAM134B and BiP **(Fig. 3k)**; scale bar = 100um.

Supplementary Fig. 5: a) Binding mode of vitexin in the catalytic pocket of BiP showing H-bond (*Dashed lines*) interactions with SER300 and ARG367 amino acid residues.

Supplementary Fig. 6: (a–d) mRNA levels of UPR markers **(a)** BiP, **(b)** XBP-1s, **(c)** ATF4 and **(d)** CHOP relative to HPRT upon Vitexin treatment during normoxia and CI-hypoxia. **(e)** Western blot of UPR markers XBp-1s, ATF4 and CHOP upon vitexin treatment during normoxia and

hypoxia. **(f)** Individual channels - confocal immunofluorescence image of MCF-7 cells treated with vitexin cultured in HE stained for FAM134B (green), BiP (purple) and nucleus – DAPI (blue).

Supplementary video 1: Live imaging of MCF-7 cell proliferation (Normoxia)

Supplementary video 2: Live imaging of MCF-7 cell proliferation in the presence of CoCl2

Supplementary video 3: Live imaging of MCF-7 cells (Normoxia) expressing WIPI-1 GFP

Supplementary video 4: Live imaging of CI-hypoxic in MCF-7 cells expressing WIPI-1 GFP

Supplementary video 5: Live imaging of normoxic MCF-7 cells expressing GFP-WIPI-1 and mCherry-ER-3

Supplementary video 6: Live imaging of CI-hpoxic MCF-7 cells expressing GFP-WIPI-1 and mCherry-ER-3

Supplementary video 7: Molecular dynamics simulation of BiP in complex with vitexin

Supplementary video 8: Live imaging of vitexin treated normoxic MCF-7 cells expressing GFP-WIPI-1 and mCherry-ER-3

Supplementary video 9: Live imaging of vitexin treated CI-hypoxic MCF-7 cells expressing GFP-WIPI-1 and mCherry-ER-3

Supplementary Table 1

	Glide Score
**Small molecule**	**BIP**
Bay872243	-4
LW6	-4.7
PX-478	-3.7
**Vitexin**	**-8.03**
YC1	-5.12
4-PBA	-4.1
GSK2656157	-5.05
STF083010	-4.7
TUDCA	-5.05
