# Supplementary figures and images for: Cancer cells adapt FAM134B/BiP mediated ER-phagy to survive hypoxic stress
Source: Cell Death Dis. 2022 Apr 18;13(4):357. doi: 10.1038/s41419-022-04813-w (PMC9016075; doi:10.1038/s41419-022-04813-w)

Figure 1

a

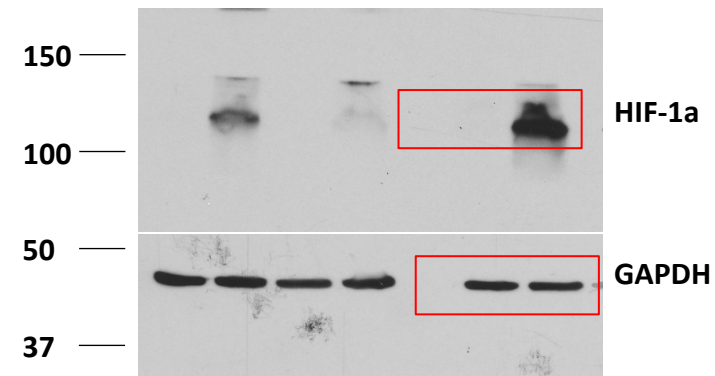

g

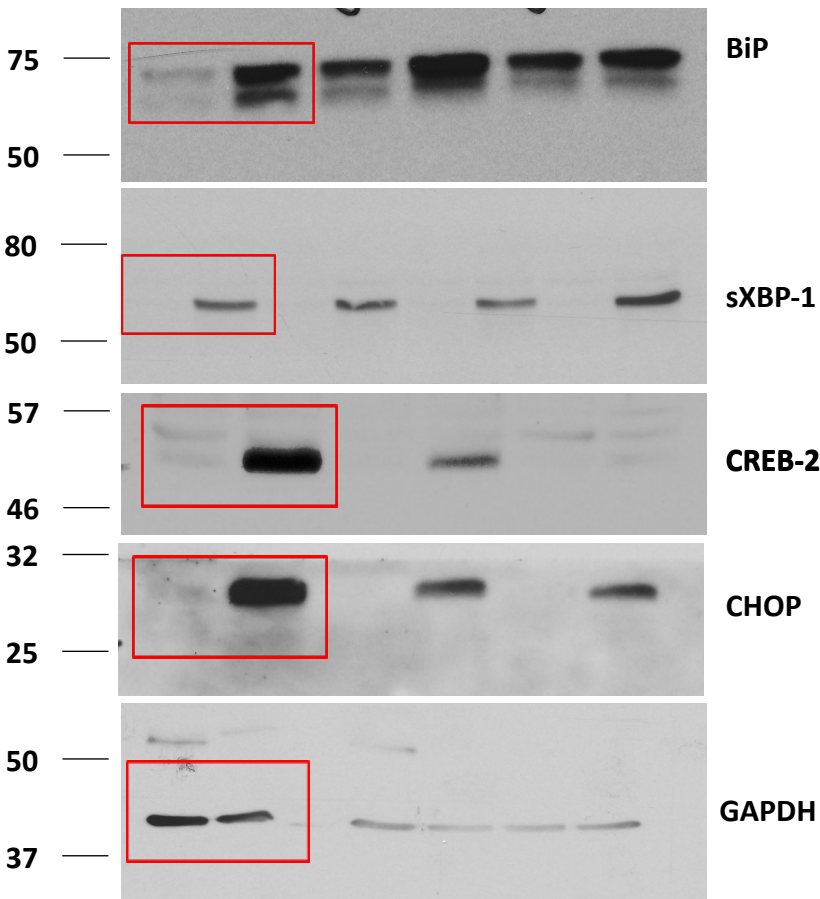

h

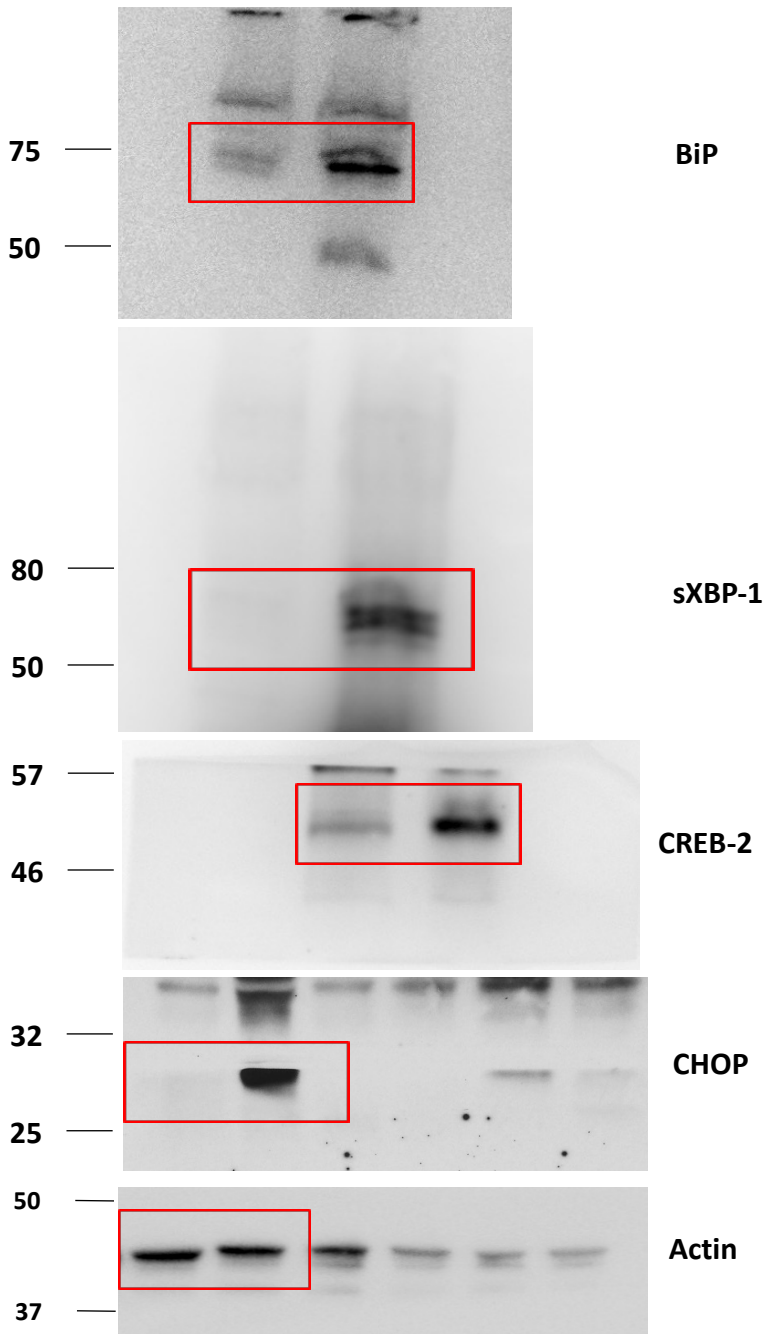

Figure 2

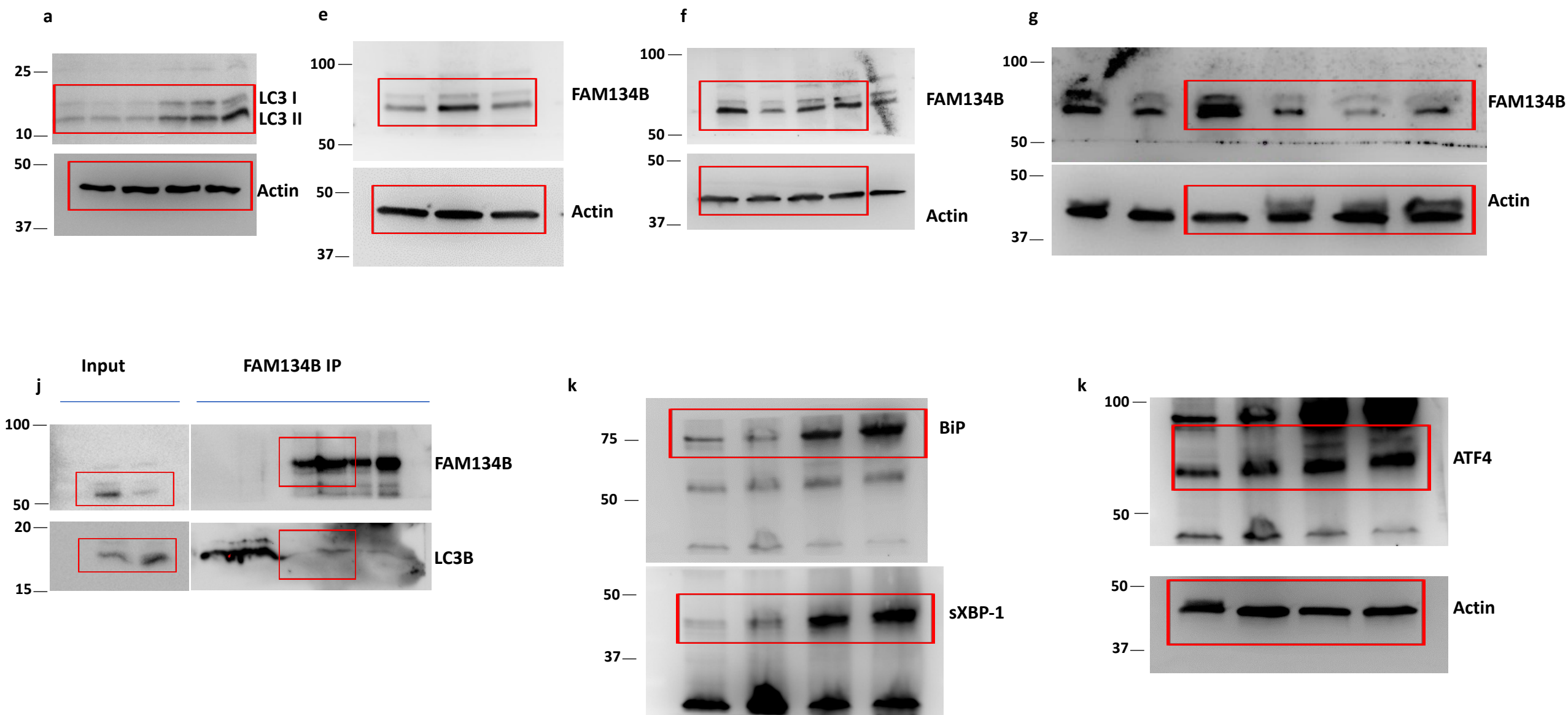

Figure 3

a

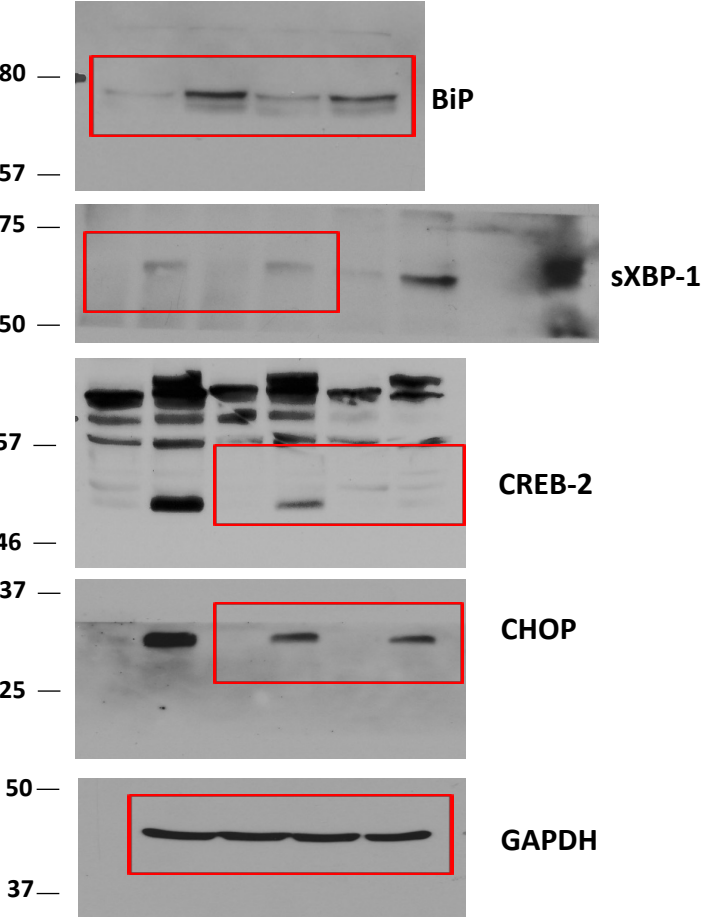

f

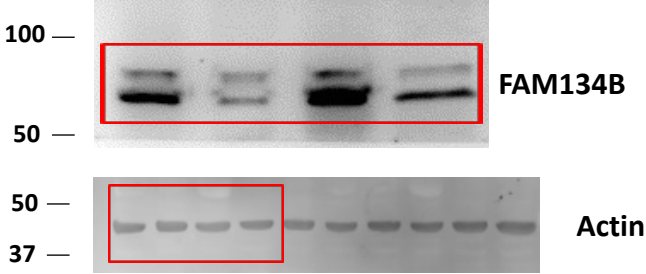

g

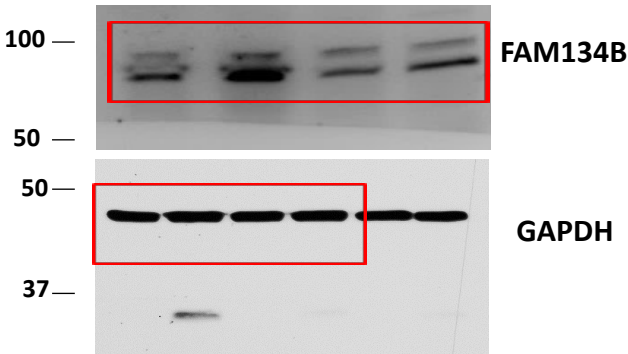

h

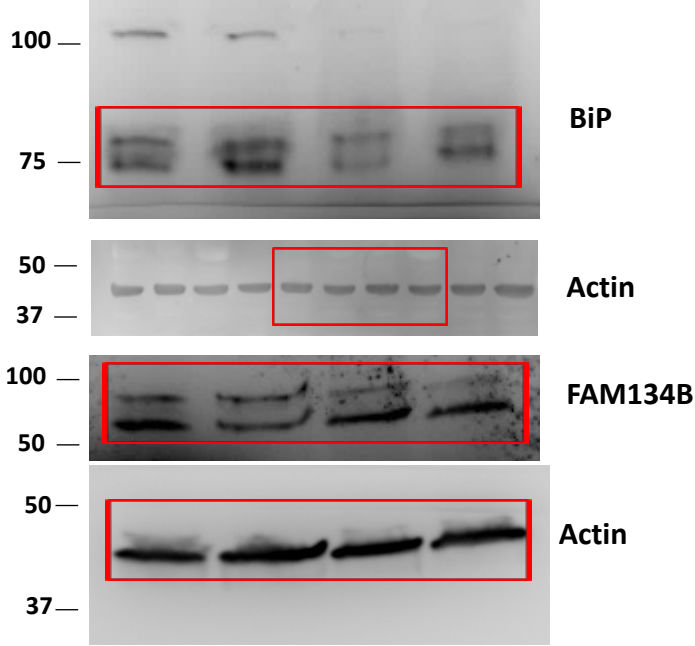

i

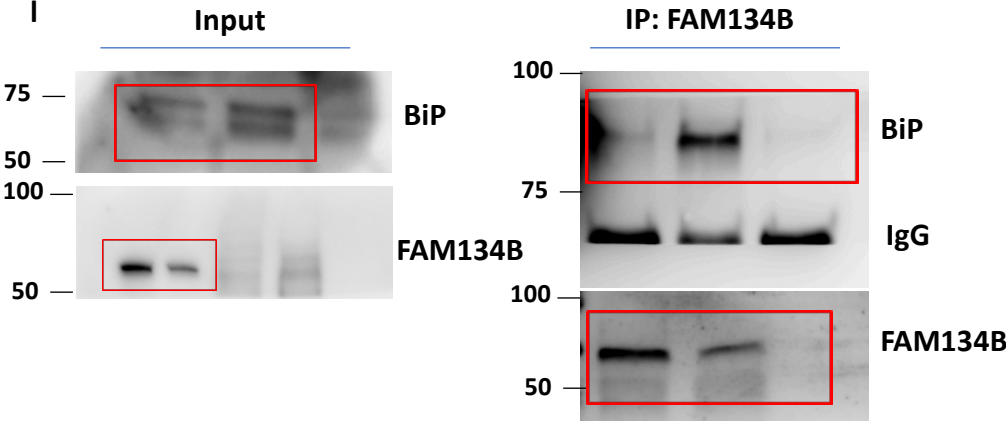

Figure 5

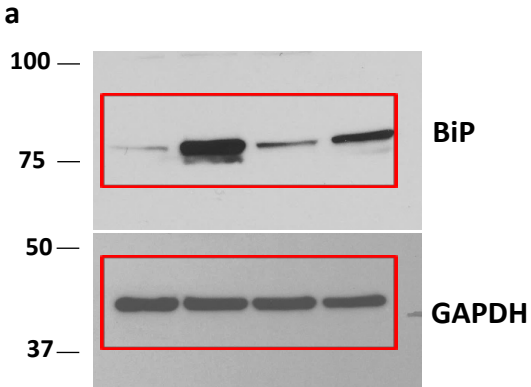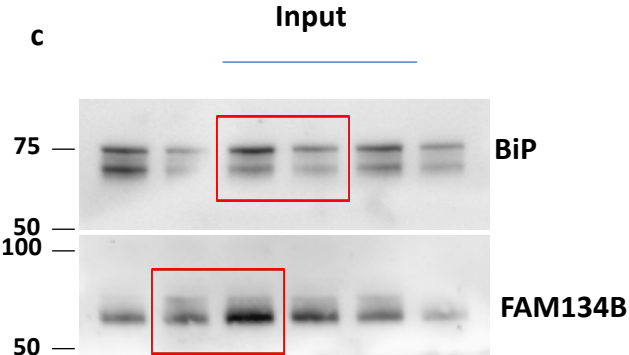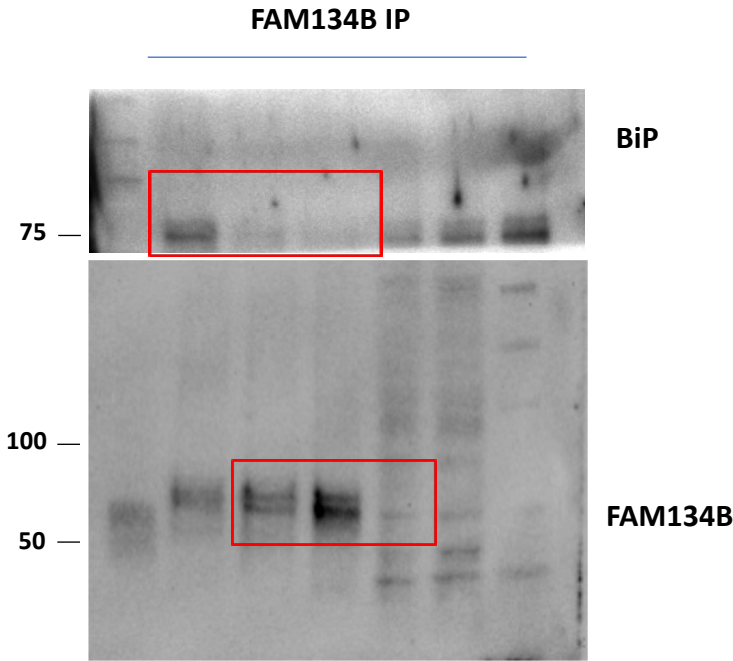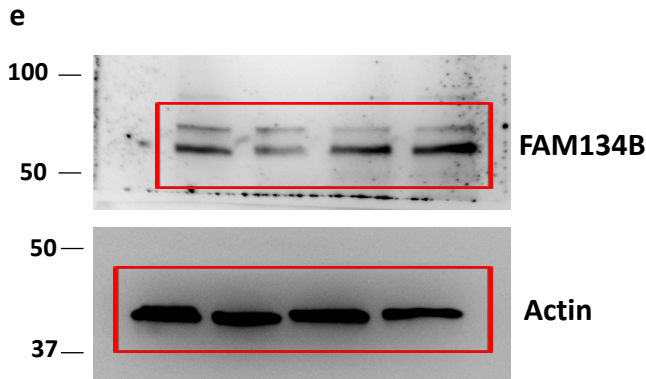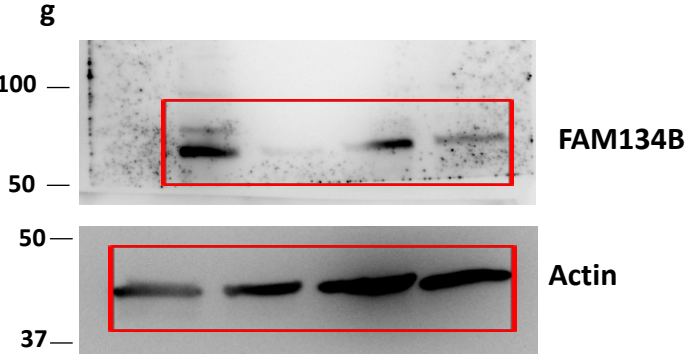

Supplement Fig 1

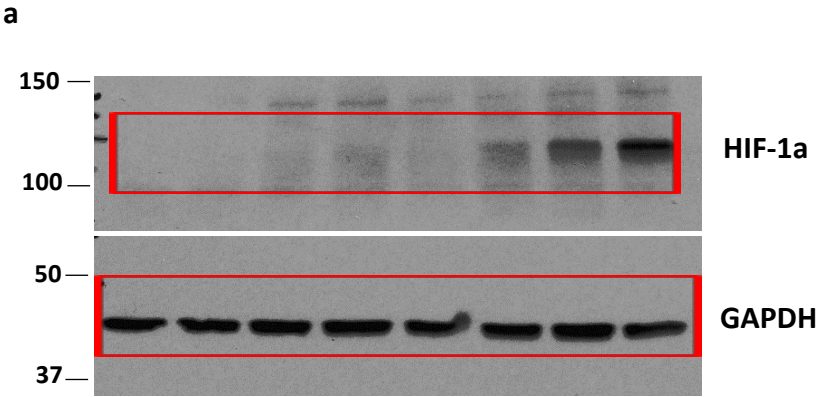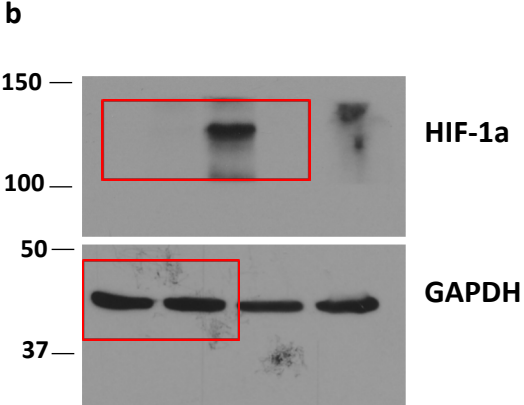

Supplement Fig 2

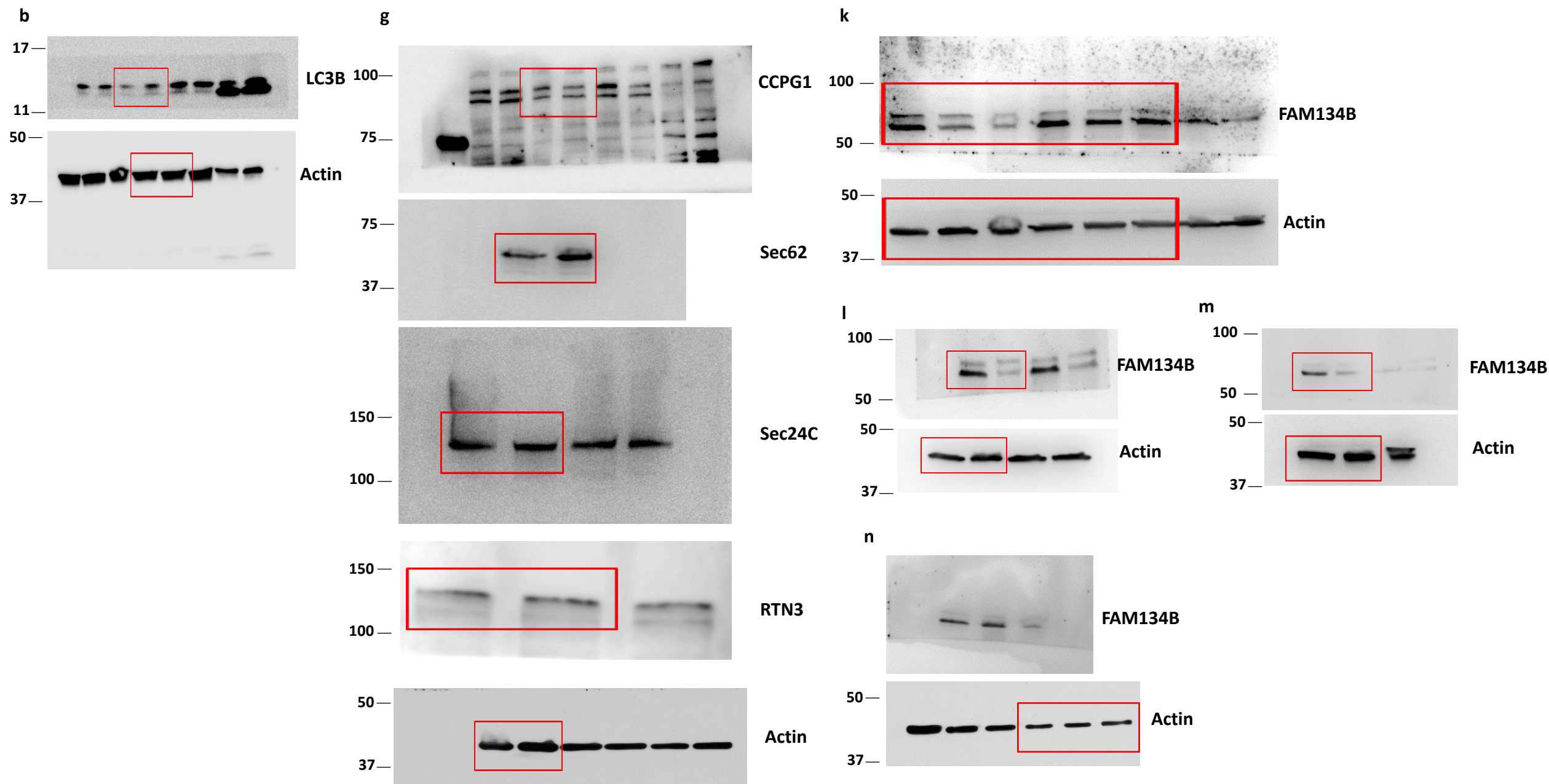

**d**

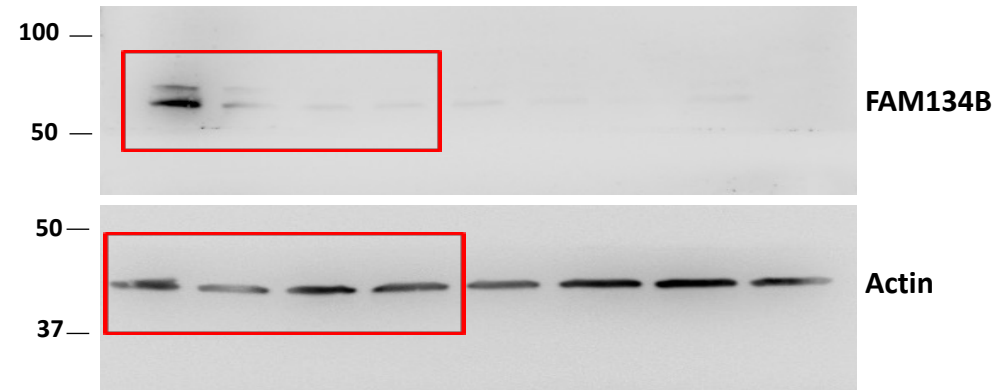

**a**

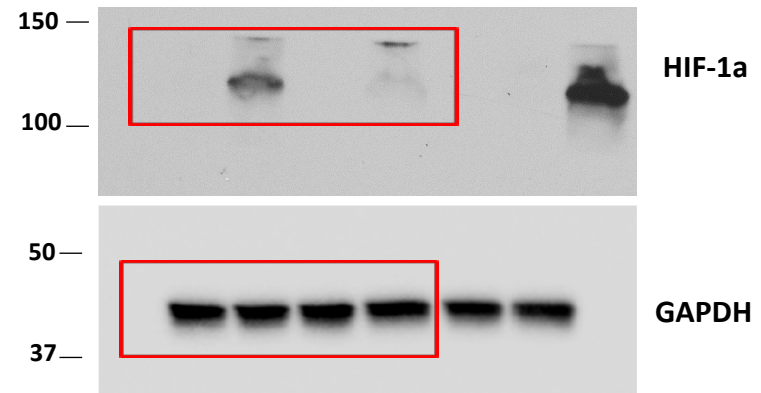

Supplement Fig 6

e

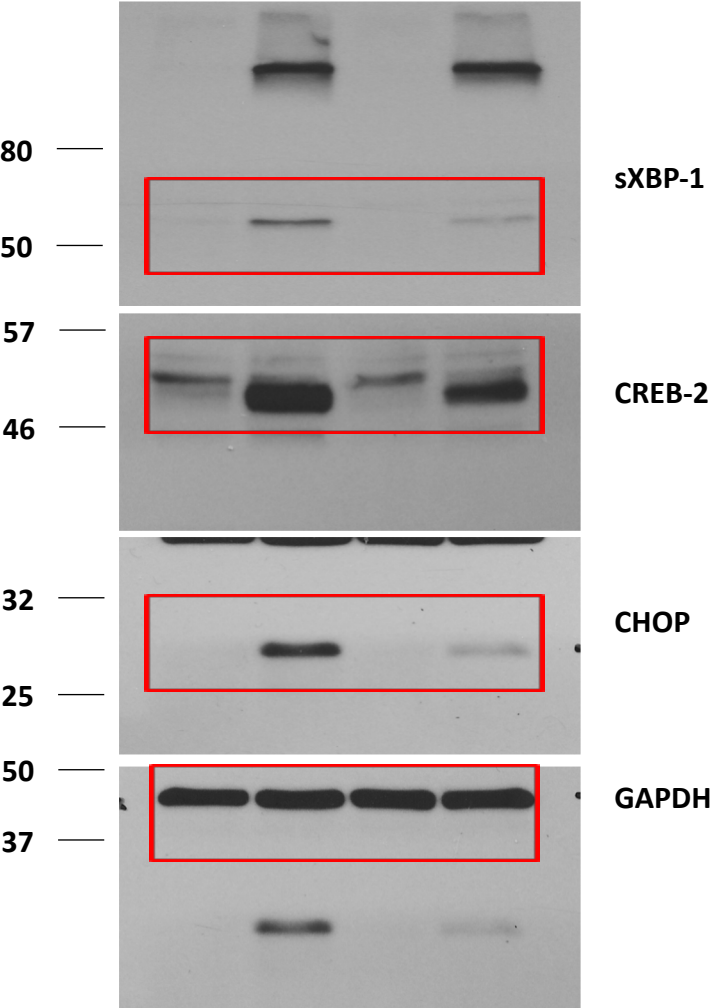

Supplement: Supplementary file 3 — Original Western Blots [file 41419_2022_4813_MOESM3_ESM.pdf]
