# Supplementary material for: Cancer cells adapt FAM134B/BiP mediated ER-phagy to survive hypoxic stress
Source: Cell Death Dis. 2022 Apr 18;13(4):357. doi: 10.1038/s41419-022-04813-w (PMC9016075; doi:10.1038/s41419-022-04813-w)
Supplement: Supplementary file 4 — CDDIS-22-0694 [file 41419_2022_4813_MOESM4_ESM.docx]

**Cancer cells adapt FAM134B/BiP mediated ER-phagy to survive hypoxic stress**

Sandhya Chipurupalli^1,2+^, Raja Ganesan^1^, Giulia Martini^3^, Luigi Mele^4^, Alessio Reggio^5^, Marianna

Esposito^5^, Elango Kannan^2^, Vigneshwaran Namasivayam^6^, Paolo Grumati^5,7^, Vincenzo Desiderio^4*^, Nirmal Robinson^1^*

1. Cellular-Stress and Immune Response Laboratory, Center for Cancer Biology, University of South Australia, Adelaide, Australia.
2. Department of Pharmacology, JSS College of Pharmacy, JSS Academy of Higher Education &

Research, Ooty, Tamil Nadu, India

1. Medical Oncology, Department of Precision Medicine, University of Campania “Luigi Vanvitelli”, Naples, Italy
2. Department of Experimental Medicine, University of Campania “Luigi Vanvitelli”, Naples,

Italy

1. Telethon Institute of Genetics and Medicine (TIGEM), Pozzuoli, Italy
2. Pharmaceutical Institute, Pharmaceutical Chemistry II, University of Bonn, Bonn, Germany
3. Department of Clinical Medicine and Surgery, University of Naples Federico II, Naples, Italy

***Running Title:*** ER-Phagy is an adaptive mechanism in cancer cells

**** Correspondence: nirmal.robinson@unisa.edu.au; vincenzo.desiderio@unicampania.it* + Current affiliation:** Departments of Pediatrics & Biochemistry and Molecular Biology, Atlantic Research Centre, Dalhousie University, Halifax, Canada.

**Abstract**

In the tumor microenvironment, cancer cells experience hypoxia resulting in the accumulation of misfolded/unfolded proteins largely in the endoplasmic reticulum (ER). Consequently, ER proteotoxicity elicits unfolded protein response (UPR) as an adaptive mechanism to resolve ER stress. In addition to canonical UPR, proteotoxicity also stimulates the selective, autophagydependent, removal of discrete ER domains loaded of misfolded proteins to further alleviate ER stress. These mechanisms can favor cancer cell growth, metastasis and long-term survival. Our investigations reveal that during hypoxia-induced ER stress, the ER-phagy receptor FAM134B targets damaged portions of ER into autophagosomes to restore ER-homeostasis in cancer cells. Loss of FAM134B in breast cancer cells results in increased ER stress and reduced cell proliferation. Mechanistically, upon sensing hypoxia-induced proteotoxic stress, the ER chaperone BiP forms a complex with FAM134B and promotes ER-phagy. To prove the translational implication of our mechanistic findings, we identified vitexin as a pharmacological agent that disrupts FAM134B-BiP complex, inhibits ER-phagy and potently suppresses breast cancer progression in vivo.

**Key Words:** hypoxia; autophagy; ER-stress; ER-phagy; FAM134B; BiP; breast cancer; vitexin **Introduction**

Cancers often encounter a characteristic microenvironment called tumor microenvironment (TME), which comprises of a chemical (pH, hypoxia, metabolite concentration) and a cellular milieu (blood vessels, immune suppressor cells, fibroblasts, extracellular matrix, stromal cells) which influences the growth of cancerous cells[1-4]. Hypoxic environment arises as a result of vascular insufficiency during the tumor expansion and progression[5]. It alters the cancer cell metabolism and contributes to therapy resistance by activating adaptive responses such as ER stress, anti-oxidative responses and autophagy[6]. Therefore hypoxia is considered a major impediment for effective anti-cancer therapy[5, 7].

ER is a multifunctional organelle with a central role for protein synthesis, modifications, and transport. The disulphide bonds that are formed during protein synthesis are independent of oxygen availability whereas, the bonds that are formed during the post-translational folding in the ER are oxygen-dependent[8]. This process is altered during hypoxia resulting in the accumulation of misfolded/unfolded proteins in the ER lumen, therefore perturbing its homeostasis. Thus, hypoxia directly impacts protein modifications in the ER leading to the activation of UPR to preserve ER homeostasis[9]. UPR is a signaling system which activates cellular responses coordinated via three key regulators – inositol-requiring enzyme 1 (IRE1), PKR-like ER kinase (PERK) and activating transcription factor 6 (ATF6)[10-13]. Binding immunoglobulin protein (BiP or glucose-regulatory protein 78 – Grp78) is a chaperone abundantly present in the ER, which transiently binds to the luminal domain of UPR receptors – IRE1, PERK and ATF6[14]. When the misfolded/unfolded proteins begin to accumulate in the ER, BiP rapidly dissociates from the three UPR signaling sensors and binds the exposed hydrophobic regions of the nascent polypeptides to facilitate proper folding[13]. In addition, UPR also induces autophagy as a key response to the stress pathway activation in cancer cells which allows them to maintain metabolic homeostasis[15-17].

Autophagy involves the sequestration of cytoplasmic components into autophagosomes which then fuse with lysosomes and degrade their contents[18]. Although, autophagy is a constitutive homeostatic mechanism which regulates intracellular recycling, it is also a major stress responsive mechanism that facilitates the removal of damaged proteins and organelles[19]. Hence, autophagy bestows tolerance to stress and sustains cell viability under hostile conditions and is considered a “double-edged sword” because of its ability to suppress tumor yet promote tumor survival under stress[19]. Despite accumulating evidences suggesting that autophagy is critical in cancer, it is still a question of intense debate and remains complex[20]. For a long time autophagy was considered a non-selective degradation pathway however, it can selectively degrade specific organelles including mitochondria (mitophagy), peroxisomes (pexophagy), ER (ER-phagy), nucleus (nucleophagy)[18] and aggregate-prone proteins (aggrephagy)[21].

ER-phagy was first described by Peter Walter's group where they demonstrated that selective engulfment of ER into autophagosomes utilize several autophagy proteins following UPR and this process is essential for the survival of cells exposed to severe ER stress [22]. ER was originally considered only as the primary source of autophagosome membranes [23] and ER membranes observed in autophagosomes was viewed as a result of bulk engulfment of cytosol [23, 24]. After the identification and characterization of specific receptors that mediate the elimination of ER through autophagosomes this type of selective autophagy was termed “ER-phagy or reticulophagy” [25]. To date, eight ER-resident proteins have been identified as selective ERphagy receptors: FAM134A[26], FAM134B[27], FAM134C[26], RTN3[28], SEC62[29], CCPG1[30], ATL3[31], TEX264[32-34] and the soluble ER-phagy receptor CALCOCO1[35] and C53[36].

Here we report that in breast cancer cells, hypoxia-induced ER stress activates ER-phagy through the ER-phagy receptor FAM134B. Upon hypoxic stress, FAM134B is included in a complex with the ER chaperone BiP to target discrete zone of ER for autophagic degradation. Inhibition of ERphagy, by silencing FAM134B, or BiP reduces breast cancer cell proliferation. To prove concrete translational implications to these mechanistic aspects, we have identified vitexin as a pharmacological inhibitor of BiP complex regulated ER-phagy that potently limits the tumor burden in a breast cancer xenograft model.

**Results**

# Hypoxia induces ER stress response and autophagy

Hypoxia is characterized by the stabilization of HIF-1a. Hence, we first investigated if HIF-1a is expressed and stabilized in our model of MCF-7 breast cancer cells upon hypoxic stress induced chemically using cobalt chloride (CoCl_2_) or growing cells in hypoxic environment (1% O_2_) for

24h. Although CoCl_2_ has been widely used as a chemical inducer of hypoxia, reports indicate that CoCl_2_ activates a complex relationship between adaptive and cell death responses[37]. We observed HIF-1a expression was concentration dependent for CI-hypoxia **(Supplementary Fig. 1a).** We chose 500µM of CoCl_2_ as an optimal concentration to induce HIF-1a **(Fig 1a)**. Exposing MCF7 cells to hypoxic environment (HE) also resulted in HIF-1a expression and stabilization

**(Supplementary Fig. 1b)**. Time lapse imaging of MCF-7 cells treated with CoCl_2_ at 500µM for

24h showed more than 2-fold increase in cell proliferation compared to the untreated cells (normoxic cells) **(Fig. 1b; Video 1 & 2)**. Similar increase in cell proliferation was also observed when cells were cultured in 1% O_2_ environment (HE) **(Fig 1c)**.

Hypoxia results in the accumulation of unfolded/misfolded proteins in the ER causing ER-stress and cancer cells adapt by activating UPR mechanisms which enable them to survive and proliferate [4, 9]. Confocal microscopy of cells expressing mCherry-ER-3 (Calreticulin-KDEL) and grown in HE showed altered ER structure compared to cells under normoxic culture conditions **(Fig. 1d).**

Furthermore, transmission electron microscopy (TEM) revealed discontinuous ER structures when

MCF7 cells were subjected to CI-hypoxia (**Fig. 1e**) or HE (**Fig. 1f**) respectively. Consistently, UPR markers BiP, spliced XBP-1 (XBP-1s), CREB-2/ATF4 and CHOP, monitored via quantitative real-time PCR (qRT-PCR) **(Supplementary Fig. 1c-f)** and western blot **(Fig. 1g)**, showed significant upregulation upon CI-hypoxia. Increased expression of UPR target proteins was also confirmed in cells grown in HE **(Fig. 1h)**.

These data suggest that hypoxia induced HIF-1a and ER-stress response correlates with increased cancer cell proliferation.

# Hypoxia induces ER-phagy to maintain ER homeostasis

It is well-known that autophagy is required for the survival of hypoxic cancer cells [38] and it assists the degradation of misfolded/unfolded proteins to reestablish ER homeostasis [4, 39]. To this end, we next investigated whether autophagy is induced in cancer cells under hypoxia. In cells subjected to CI-hypoxia **(Fig. 2a and Supplementary Fig.2a)** and HE **(Supplementary Fig. 2b,c)**, LC3B showed increased conversion to its lipidated form (LC3II) compared to the normoxic cells. Moreover, inhibition of lysosomal activity using concanamycin A led to further increase in the accumulation of LC3II in cells treated with CoCl_2_ **(Fig. 2a and Supplementary Fig. 2a)**.

Remarkably, MCF7 cells in CI or HE conditions_,_ showed increased distribution of WIPI-1 compared to normoxic cells **Fig. 2b and Supplementary Fig. 2d-f**). Similarly, LC3B puncta were also observed in the hypoxic regions of MMTV-pyMT mouse breast cancer tissue characterized by HIF-1a in the nucleus **(Fig. 2c)**. During autophagy induction, damaged organelles are selectively targeted into autophagosomes for degradation. TEM of MCF7 cells subjected to CI and HE-hypoxia showed accumulation of damaged ER within autophagosomes **(Fig. 2d)**. WIPI-1 was also found to colocalize with calnexin in MCF7 cells subjected to CI-hypoxia **(Fig 2b & Supplementary Fig. 2d)** and HE (**Supplementary Fig. 2e, f**). Consistent results were obtained from time lapse imaging of CI-hypoxic cells **(Video 5&6)**. TEM and co-localization of WIPI-1 with the ER suggested that damaged ER is possibly engulfed by autophagosomes to mitigate ERstress caused by hypoxia. Therefore, we questioned if the recently described ER-selective autophagy (ER-phagy)[40]^,^[41] was involved in the removal of damaged ER. We investigated the steady state levels of ER-phagy-specific receptors such as FAM134B, RTN3, SEC62, CCPG1 and the COPII subunit SEC24C which have been shown to target ER for autophagosomal degradation[42]. We did not observe any change in the protein abundance of CCPG1, RTN3 and SEC24C except a modest increase in SEC62 **(Supplementary Fig. 2g)**. In contrast, FAM134B significantly decreased upon CI-hypoxia and in cells grown in HE **(Fig 2e and Supplementary Fig. 2h)**. However, removal of hypoxic stress by replacing CoCl_2_ with medium without CoCl_2,_ restored the steady state levels of FAM134B levels **(Fig 2f and Supplementary Fig. 2i)**. We found that the decline in FAM134B was due to lysosomal degradation as inhibition of lysosomal activity using concanamycin A prevented FAM134B degradation during HE hypoxia **(Fig. 2g and Supplementary Fig. 2j)** and CI-hypoxia **(Supplementary Fig. 2k)**. We also observed a similar degradation of FAM134B in U251 glioblastoma cells **(Supplementary Fig. 2l)** and C32 melanoma cells (**Supplementary Fig. 2m)** indicating ER-phagy as a general mechanism that cancer cells exploit to counteract hypoxia-induced stress. In addition, we observed FAM134B degradation when ER-stress was induced using tunicamycin in MCF7 cells, but it did not occur when cells were starved by depleting serum in the culture medium **(Supplementary Fig. 2n)**. This suggests that, in MCF7 cells, FAM134B degradation occurs only upon ER-stress but not specific to hypoxia induced ER-stress. Furthermore, we observed co-localization between LC3 and FAM134B in hypoxic cells expressing HA-tagged FAM134B **(Fig. 2h and Supplementary Fig. 3a,b)** and in MMTV-pyMT mouse breast cancer tissue sections **(Fig. 2i and Supplementary Fig. 3c)**. Moreover, LC3B co-immunoprecipitated with endogenous FAM134B under hypoxic

condition while, there was little interaction between FAM134B and LC3B in control cells, strongly supporting an increase in ER-phagy upon hypoxia **(Fig. 2j)**. Having found that hypoxia induces ER-phagy, we next examined if FAM134B-dependent ER-phagy contributes to ER-stress and cell proliferation upon hypoxia. As previously shown, CI-hypoxia increased UPR but, knocking down

*FAM134B* **(Supplementary Fig. 3d)** further enhanced the induction of UPR proteins **(Fig. 2k and**

**Supplementary Fig. 3e-g)** and transcription of UPR genes BiP, XBP-1s and ATF4/CREB2 **(Supplementary Fig. 3h-j)** meaning increased ER-stress. Silencing *FAM134B* modestly reduced cell proliferation under normoxic conditions but was highly reduced under hypoxic conditions **(Fig. 2l)**.

These observations indicate that hypoxia induces ER-phagy to overcome ER-stress and that FAM134B-dependent ER-phagy is vital for cancer cells to proliferate under hypoxic stress.

# Hypoxia induced ER-phagy is BiP dependent

Since we deciphered that hypoxia leads to activation of UPR and FAM134B dependent ER-phagy, we asked whether this circuit is HIF-1a dependent. Although depletion of HIF-1a (**Supplementary Fig. 4a**) limited hypoxia-induced UPR **(Fig. 3a-e)**, degradation of FAM134B was not affected in cells subjected to CI-hypoxia **(Fig. 3f and Supplementary Fig. 4b)** and HE **(Fig. 3g and Supplementary Fig. 4c)**. This suggests that hypoxia-induced ER-phagy is independent of HIF-1a but, triggered by an alternative pathway most likely connected to the presence of misfolded ER proteins. FAM134B lacks an intraluminal domain therefore, we wondered if it indirectly senses the accumulation of unfolded/misfolded proteins likely through an ER stress chaperone. We observed that, silencing BiP prevented the degradation of FAM134B during hypoxia **(Fig 3h and Supplementary Fig. 4d)** signifying that ER-phagy is BiP-dependent during hypoxic stress in MCF7 cells. BiP was also found to colocalize with FAM134B when cells were subjected to HE (**Fig. 3i and Supplementary Fig. 4e).** Consistently, BiP co-localized with FAM134B in MMTV-pyMT mouse breast cancer tissue sections **(Fig. 3j and Supplementary Fig. 4f)** and in human breast cancer tissues **(Fig. 3k and Supplementary Fig. 4g)**. Of note, BiP co-immunoprecipitated with endogenous FAM134B when MCF7 cells were subjected to hypoxia **(Fig. 3l)**. In addition, silencing *BiP* significantly reduced the proliferation of MCF7 breast cancer cells under normoxic and hypoxic conditions (**Fig. 3m)**.

Taken together, these data show that ER-phagy is a specific response to ER-stress and is coregulated by BiP and FAM134B.

# In-Silico molecular docking studies identified Vitexin as a potential BiP inhibitor

Above shown results revealed that ER-phagy alleviates ER-Stress response and facilitates the survival and progression of hypoxic cells. Hence, the selective disruption of this axis may have a tremendous clinical value. To this end, we retrieved the high-resolution X-ray crystal structure of the protein from the Protein Data Bank (PDB ID: 5F0X.pdb, Resolution: 1.6 Å). We next performed molecular docking studies using Schrödinger Suite 2015-3. A small set of small molecules library was docked onto the ATP/ADP binding site, which is occupied by the subdomains Ia, Ib and IIa of the protein [43] and identified an apigenin flavone glucoside, vitexin as a potential molecule targeting BiP (Docking scores are shown in Supplement Table 1). Vitexin showed lowest glide score towards BiP i.e., -8.3Kcal/mol **(Supplement Table 1)**. The putative binding mode of vitexin and important residues in the binding site of the BiP are shown in **Fig. 4a-b**. The binding site comprises a higher number of charged and polar residues. Vitexin was bound inside the binding site by two strong hydrogen bonding interactions between OH of the glucoside and the side chain of amino acid residues N389 and R367. The keto group of the flavanone moiety forms hydrogen bond interaction with S300 and arene-hydrogen interaction with R297 **(Fig 4c)**. These predicted interactions of vitexin resulted in top rank based on the docking score meaning higher binding affinity when compared to the other molecules screened against BiP. As a next step, we performed molecular dynamics simulations of the BiP-vitexin complex for explaining the stability of the predicted binding pose in the binding site of the protein and compared with the simulations of the protein structure without the ligand inside the binding site. The calculated root mean square deviation (RMSD) values of the Cα atoms of the complex and the apo structure rapidly reached an equilibrium state with approximately 1 Å deviation from the first frame of 100 ns simulations **(Fig. 4d)**. The visual analysis of the trajectories shows that vitexin was anchored inside the binding site with the interaction pattern identified from the docking studies **(Supplementary Fig. 5a, Supplementary video Supplementary Video 7)**. It maintains the key hydrogen bond interaction with the three residues (N389, R367 and S300) and possible arene interaction with R297. The root mean square fluctuation (RMSF) value of the protein showed a profile with large fluctuations in the Cα atoms are more stabilized in the BiP in complex with vitexin when compared to the protein without the binding of vitexin in the binding site **(Fig. 4e)**. Based on these findings using molecular docking and molecular dynamics approaches, we

subjected vitexin for further in-vitro validation.

# Vitexin prevents FAM134B-BiP interaction and inhibits ER-phagy

Since our *in silico* molecular dynamics studies deciphered vitexin as a potential inhibitor of BiP, we next examined BiP protein levels in hypoxic cells treated with vitexin. Consistently, vitexin treatment downregulated BiP during hypoxia **(Fig. 5a-5b)**. Indeed, treatment with vitexin also downregulated CI-hypoxia induced UPR at the mRNA **(Supplementary Fig. 6a-d)** and protein levels **(Supplementary Fig. 6e)**. We then asked if treatment with vitexin prevented the interaction of FAM134B with BiP during hypoxia. Strikingly, BiP did not coimmunoprecipitate with FAM134B upon vitexin treatment in hypoxic cells **(Fig. 5c)** and was found to less colocalize with BiP **(Fig. 5d and supplementary Fig. 6d)**. Furthermore, immunoblot analysis revealed that vitexin prevents autophagosome degradation of FAM134B during CI-hypoxia **(Fig. 5e-f)** and HEhypoxia **(Fig. 5g-h)**. Consistently, time-lapse imaging of vitexin-treated cells expressing GFPWIPI-1 and mCherry-ER-3 exhibited accumulation of autophagosomes. Thus, autophagic flux and proteins turnover were inhibited leading to cell death **(Video 8 & 9)**.

Collectively, these data confirm that vitexin blocks ER-phagy by inhibiting BiP from forming a complex with FAM134B

# Vitexin reduces cancer cell proliferation and tumor burden in breast cancer xenograft mouse model

Having shown that vitexin inhibits ER-phagy, we surmised that it could inhibit cancer cell proliferation under hypoxic stress. As expected, vitexin treatment effectively prevented the increase in cell proliferation upon hypoxic stimuli **(Fig. 6a)**. As we propose that ER-phagy resolves ER-stress, we also explored if vitexin can synergistically inhibit cancer cell growth with an ERstress inducer tunicamycin. We observed that vitexin and tunicamycin synergistically inhibited cancer cell growth **(Fig. 6b)**. We next asked if vitexin can reduce tumor burden in female balb/c athymic (nuþ/nuþ) mice xenografted with MCF7 cells. We found that after 13, 17 and 21 days of vitexin treatment, tumor volume was significantly reduced compared to the vehicle treated mice **(Fig. 6c,d)**.

Taken together, we could conclude that vitexin shows a higher therapeutic potential in treating breast cancer.

# Discussion

Identification of receptors that specifically target damaged organelles and proteins into autophagosomes for degradation assists in discriminating from functional organelles. Recently, specific receptors have been identified to target damaged or excess parts of ER into autophagosomes for elimination and this process has been termed ER-phagy[44]. Here, we report that cancer cells undergo ER-phagy regulated by a complex including FAM134B and BiP when they are subjected to hypoxic stress which helps the cells to mitigate ER-stress and promote cell proliferation **(Fig 6e)**.

Numerous studies have confirmed that the TME promotes cancer progression due to the ability of tumor cells to overcome stress and survive in the hostile microenvironment. Hallmark of TME is diminished levels of oxygen (less than 2%) which is essential for a cell to meet its bioenergetic needs [45]. Hypoxic conditions can be mimicked in the laboratory by either growing the cells in a hypoxic incubator with reduced levels of oxygen or treating cells with CoCl_2_ [46]. Cells undergoing hypoxic stress stabilize HIF-1a which we could observe in cells subjected to CoCl_2_ treatment and those grown in a reduced Oxygen environment. Oxygen is not only required to meet the metabolic needs of the cells but is also essential for protein disulphide bond formation during protein-folding in the ER[8]. However, hypoxia disrupts protein folding resulting in the accumulation of unfolded/misfolded proteins causing ER-stress and cell death [47]. Cancer cells alleviate ER-stress by activating UPR characterized by the expression of BiP, XBP1-s, CHOP and ATF4 [48, 49]. Our data also suggest that UPR could be regulated by HIF-1a in hypoxic cells as depletion of HIF-1a abrogates the expression of UPR proteins while, the molecular mechanism by which HIF-1a regulates UPR remains to be investigated.

UPR has been shown to induce autophagy which helps in the removal of misfolded proteins and damaged organelles thus preventing DNA damage and cancer progression. On the contrary, when the cancer cells undergo nutrient starvation or hypoxia, autophagy acts as a quality control mechanism to overcome the stress and survive[19]. Consistently, autophagy is increased when cancer cells are subjected to hypoxic stress as determined by LC3I to II conversion. Autophagy encompasses autophagosome formation marked by LC3 conversion and degradation of the cargo by fusion with the lysosomes. During general/macroautophagy, LIR (LC3-interacting region) motif containing adaptor proteins recognize ubiquitinated substrates and target them for autophagic degradation by binding to LC3[50, 51]. Mounting evidence suggest that autophagy selectively degrades damaged organelles to maintain cellular homeostasis. In line with this, hypoxia has been shown to target mitochondria (mitophagy) [52] and peroxisomes (pexophagy) [53] for autophagic degradation.

As hypoxia causes damage to ER, a failure to restore ER structure and homeostasis could be lethal to cells. Restoration of ER is achieved by the removal of excess and damaged ER caused by ERstress. Recent evidences suggest that ER can be selectively targeted for lysosomal degradation by ER-phagy[54]. UPR has also been linked to the activation of ER-phagy however, no direct evidence linking hypoxia and ER-phagy has been reported thus far. Our data show that ER is selectively targeted for degradation in autophagosomes during hypoxia. Several proteins containing LIR motif have been shown to function as ER-phagy receptors, which have been shown to recruit subregions of the ER into autophagosomes[42]. For instance, FAM134B is localized on the edges of the ER sheets where protein synthesis and folding occurs, and has a clear role in maintaining ER volume and proteostasis[27, 28]. The reticulon homology domain (RHD) of FAM134B aids in the fragmentation of ER and LIR motif sequesters the fragmented ER into autophagosomes[25]. Hypoxia resulted in the colocalization of FAM134B with autophagosomal marker LC3 and was subsequently degraded in lysosomes indicating that cancer cells remove damaged ER by activating FAM134B-dependent ER-phagy. In addition, silencing of FAM134B during UPR results in cell death[27]. Consistently, hypoxia stimulated ER-stress reduces the viability of FAM134B-depleted breast cancer cells. We also observed a modest increase in SEC62 which has been shown to specifically regulate the recovery of ER[29, 55] but was not targeted for autophagic degradation during hypoxia. However, we cannot exclude the involvement of other ER-phagy receptors, which will be explored in the future. Notably, hypoxia upregulates FAM134B expression in chronic myeloid leukemia (CML) cells and is correlated with pro-survival[56]. It is also speculated that its upregulation is HIF-1a dependent[57]. However, silencing of HIF-1a did not alter the relative steady state levels of FAM134B neither during hypoxia compared to normoxia.

FAM134B lacks intraluminal domains hence the question that remains to be answered is, how does FAM134B detect ER-stress or physiological changes within the ER? It is likely that it cooperates with accessory proteins to detect ER stress [25]. BiP and Calnexin are the two major chaperone systems in the ER lumen[58, 59] and recently it has been reported that FAM134B cooperates with Calnexin which possesses a luminal chaperone domain to sense and remove misfolded procollagen [60]. BiP is a chaperone which senses accumulation of misfolded proteins in the ER resulting in the release of BiP from the UPR proteins and also chaperones the folding of accumulated proteins [13]. We noticed that BiP co-immunoprecipitated with FAM134B in hypoxic cells and colocalizes in the breast cancer tissues. Additionally, depletion of BiP prevented

FAM134B-dependent ER-phagy and stalled the proliferation of cancer cells subjected to hypoxia. Even if FAM134B does not have an ER luminal domain and BiP is an ER luminal chaperone, the two proteins seem to be connected in a common complex. Future experiments will be addressed to fully clarify the biochemistry of this complex and what are the other proteins that likely bridge FAM134B and BiP during the hypoxia stress.

Though ER-phagy has been speculated to be involved in various pathologies including cancer [61], therapeutic options to target ER-phagy has not been exploited yet. Having found that depletion of BiP prevents ER-phagy and cancer cell proliferation, we explored to pharmacologically target BiPdependent ER-phagy. *In-silico* analysis identified vitexin, a plant derived flavone C-glycoside (apigenin-8-C-β-d-glucopyranoside) [62-64] as an inhibitor of BiP. Although several studies have reported the therapeutic potential of vitexin in treating various medical disorders including cancer, the precise molecular target remains unresolved. We show that vitexin not only abrogates BiP dependent UPR but also inhibits FAM134B-dependent ER-phagy assisted by BiP. Decrease in UPR downstream of IRE-1 and PERK in vitexin treated cells could suggest that ER-stress is mitigated by vitexin. However, vitexin induces UPR in normoxic cells and prevents the proliferation of cancer cells. This indicates that vitexin induces UPR as it inhibits ER-phagy, which could potentially relieve the ER-stress. It also suggests that vitexin not only prevents BiP from binding to FAM134B but also prevents the ability of BiP to chaperone unfolded/misfolded proteins. Furthermore, induction of ER-stress with tunicamycin combined with ER-phagy inhibition using vitexin synergistically stunted cell proliferation. Therefore, we surmise that unresolved ER-stress is detrimental to cell survival and proliferation.

Disruption of ER and ER homeostasis could lead to the death of cells and cause disease pathologies. However, during the late stages of cancer when cancer cells are under various metabolic stresses including hypoxia, they adapt mechanisms such as ER-selective autophagy to overcome the damage to ER and the associated cellular processes. Therefore, targeting such adaptive mechanisms is a potential way forward to treat cancer. Our data reported here unveils FAM134B-BiP complex-mediated ER-phagy as a novel mechanism by which cancer cells prevail over hypoxia induced proteotoxic stress and targeting ER-phagy machinery as a prospective therapeutic strategy to treat cancer.

**Materials and Methods**

# Cell culture

MCF-7 cells were cultured in Dulbecco's Modified Eagle Medium (DMEM), C32 cells and U251 cells were cultured in RPMI medium supplemented with 10% fetal bovine serum and incubated at 37^o^C, 5% CO_2_. For hypoxia experiments, cells were incubated in the hypoxia incubator at low oxygen levels i.e. 1% O_2_.

# Drugs and treatments

CoCl_2_ 0.1M readymade solution (Cat No. 15862) and vitexin (CAS No. 3681-93-4) were procured from Sigma-Aldrich. CoCl_2_ and vitexin were used at a concentration of 500µM and 20µM respectively. Concanamycin A was purchased from Sigma Aldrich and used at a concentration of 100nM.

# Immunoblotting

MCF-7 cells were lysed in radioimmunoprecipitation assay (RIPA) buffer supplemented with protease and phosphatase inhibitors. Protein concentrations were estimated using Pierce BCA Protein assay kit (Thermo Fisher Scientific), as per the instructions. Equal amounts of proteins were separated on either 10% SDS/PAGE gels or 4-20% Mini-PROTEAN TGX Stain-Free Gels (#4568094, Bio-rad). Proteins were then transferred onto PVDF membranes and probed with the following antibodies: HIF-1α (D2U3T) (#14179, Cell Signaling Technology), BiP (C50B12)

(#3177, Cell Signaling technology), CREB-2 (SC-200, Santacruz), CHOP (#2895, Cell Signaling

Technology), LC3B (#83506, Cell Signaling Technology), ER Stress Antibody Sampler Kit

(#9956, Cell Signaling Technology), Sec24C (#14676, Cell Signaling Technology), Lamin B1

(#12586, Cell Signaling Technology), CCPG1 (ab150465, Abcam), Sec62 (ab137022, Abcam), FAM134B (#61011, Cell Signaling Technology) and anti-FAM134B polyclonal antibody (a kind gift from Ivan Dikic), RTN3 (# PA578316 Thermo Fisher Scientific), Normal Rabbit IgG (#2729, Cell Signaling Technology), XBP-1s (#12782, Cell Signaling Technology), SQSTM1/p62 (#5114,

Cell Signaling Technology). Beta actin, calnexin (#2679, Cell Signaling Technology) or GAPDH (sc-32233, Santacruz) were used as loading controls. After incubation with secondary horseradish peroxidase (HRP)-conjugated antibodies, the blots were washed and developed using enhanced chemiluminescence reagent in the Chemidoc MP or ImageQuant LAS4000.

# Immunofluorescence staining and confocal microscopy

MCF-7 cells grown on the glass coverslips were treated with CoCl2 and vitexin for 16-24 h and fixed with 4% formaldehyde in PBS for 15 min at RT. The cells were then permeabilized with 0.3% Triton X-100 in PBS for 5 min and blocked with 3% BSA for 1 h at RT. The cells were incubated overnight with primary antibody against HIF-1a, BiP, CREB-2, CHOP, LC3B, calnexin and FAM134B at 4^o^C. After overnight incubation, the cells were washed with PBS and incubated with either Alexa Fluor 594-conjugated goat anti-rabbit/anti-mouse or Alexa Fluor 488-conjugated goat anti-rabbit/anti-mouse secondary antibody for 1h at RT in the dark. The cells were then washed, and coverslips were mounted using ProLong Diamond antifade containing DAPI to stain the nuclei. Staining of mouse and human breast cancer tissues was performed after antigen retrieval using 0.01M Citrate buffer pH 6.0. The slides were imaged under Leica SP8 confocal or Leica THUNDER imager. Human breast cancer tissues were obtained with consent from the patients which was approved by the Ethical committee of the University of Campania “Luigi Vanvitelli” (Prot. 71-13/2/2129).

# Quantitative real-time PCR

Total RNA from MCF-7 cells (1x10^6^ cells/well) was isolated using RNeasy Mini kit (74106;

Qiagen), and 500ng cDNA was synthesized with random hexamers by reverse transcription (SuperScript III; 18080; Invitrogen). 20µL of PCR reactions contained10ng cDNA, 0.4 µmol/liter of each forward and reverse primer, and master mix (SsoFast EvaGreen Supermix; 1725201; BioRad). Real-time PCR was performed under the following conditions: initial denaturation step at 95^o^C for 2 min and 40 cycles at 95^o^C for 5 s and 60^o^C for 15 s, followed by a denaturation step at95^o^C for 60 s and a subsequent melt curve analysis to check amplification specificity. Results were analyzed by the comparative threshold cycle method with hypoxanthine-guanine phospho ribosyl transferase (*HPRT*) as the endogenous reference gene for all reactions. The relative mRNA levels of untreated samples were used as normalized controls for the CoCl2 and vitexin treated samples. All reactions were performed in triplicate and a non-template control was included in all experiments to exclude DNA contamination. Primer sequences are listed in Table 1. **Table 1: Primers used in the study**

| **S.No** | **Primer** | **Sequence (5' to 3')** |
| --- | --- | --- |
| 1 | hsXBP1-For | CTG AGT CCG AAT CAG GTG CAG |
| 2 | hsXBP1-Rev | ATC CAT GGG GAG ATG TTC TGG |
| 3 | hATF4-For | GTT CTC CAG CGA CAA GGC TA |
| 4 | hATF4-Rev | ATC CTG CTT GCT GTT GTT GG |
| 5 | hCHOP-For | AGA ACC AGG AAA CGG AAA CAG A |
| 6 | hCHOP-Rev | TCT CCT TCA TGC GCT GCT TT |
| 7 | hBIP-For | TGT TCA ACC AAT TAT CAG CAA ACT |
| 8 | hBIP-Rev | TTC TGC TGT ATC CTC TTC ACC AGT |

# Time-Lapse imaging for cell proliferation

MCF-7 cells were seeded (2x10^4^ cells/well) in the ibidi µ slide 8 well (cat no.80827) suitable for live cell imaging in the CellVoyager CV1000 confocal imaging system (Yokogawa). A day after, the cells were treated with appropriate concentrations of CoCl_2_ and vitexin and set up for timelapse imaging over 24 h duration at an interval of 20min.

# Time-Lapse Imaging for ER-phagy

MCF-7 cells were transfected with mCherry-ER-3 and GFP-WIPI-1 plasmids and seeded (2x10^4^ cells/well) in the Nunc™ Lab-Tek™ II Chamber Slide (cat no. 154526) suitable for live imaging. The next day, following treatment with CoCl2 and vitexin cells were set up for time-lapse imaging in the Leica SP8 confocal or Leica THUNDER imager over 24 h duration at an interval of 20min.

The data was processed and analyzed using imageJ software.

# siRNA and Plasmid transfection experiments

MCF-7 cells (0.5x10^6^ cells/well) were incubated with either 100nM nontargeting siRNA (SRCL000-005; Silencer™ Select) or 100nM siRNA specific for HIF-1a (HIF1a; L-004018-00-0005; Dharmacon), BiP (siRNA ID: s29012; Silencer™ Select)and FAM134B (siRNA ID: s29012, s29013; Silencer™ Select) together with the transfection reagent Lipofectamine 3000 (L3000-008; Invitrogen) for 48 h according to the manufacturer’s instructions. Knockdown efficiency was assessed by Western blot analysis using antibodies against HIF-1a, BiP and FAM134B respectively. mCherry-ER-3was a gift from Michael Davidson (Addgene plasmid # 55041; http://n2t.net/addgene:55041 ; RRID:Addgene_55041) andpMXs-IPGFP-WIPI-1[65]was a gift from Noboru Mizushima (Addgene plasmid # 38272 ; http://n2t.net/addgene:38272 ;

RRID:Addgene_38272) plasmids were transiently transfected into the MCF-7 cells for 48h using Lipofectamine 3000.

# Immunoprecipitation

MCF-7 cells (5.0 × 10^6^ cells/well) were lysed with RIPA buffer containing protease and phosphatase inhibitors. After preclearing the cell lysate with protein A/G agarose magnetic beads (16-663; Millipore) for 1 h, beads were removed by placing the tube on a magnetic rack. The whole cell lysate (
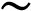
1000 µg of protein) was incubated overnight at 4°Cwith 4 µg of an antibody against FAM134B. Protein A/G agarose beads were added again and incubated for an additional 1 h at room temperature. The immunoprecipitated proteins along with the agarose beads were collected by placing the tube on a magnetic rack. The collected beads were washed three times with RIPA buffer. The washed samples were mixed with SDS-PAGE sample loading buffer, boiled, and resolved on a 10% SDS–polyacrylamide gel. The respective proteins precipitated were probed for specific antibodies for immunoblot analysis.

# Crystal Violet cell viability assay

MCF-7cells were cultured in 96-well plate at a density of 1x10^4^ cells/well. Some wells were kept without cells to serve as control for non-specific binding of the crystal violet. After 16-24 h, medium was aspirated and added 100µL of fresh medium supplemented with appropriate concentrations of drugs (CoCl_2_ and vitexin) and incubated for 24 h at 37^o^C in standard culture conditions. After 24 h of incubation, wells were gently washed twice with water and incubated with 50µL 0.5% crystal violet staining solution for 20min at room temperature on a bench rocker at a frequency of 20 oscillations per minute. Then the plate was air-dried without the lid for 2 h at room temperature and incubated with 200µL methanol for 20min at room temperature on a bench rocker at a frequency of 20 oscillations per minute. Absorbance was measured at 595nm using a microplate reader (Bio Tek^TM^ EPOCH). ***In-Silico* molecular docking studies**

**Ligand preparation:** The 2D structures of the selected ligands were drawn using ChemSketch software (https://chemsketch.en.softonic.com/). The ligands were prepared using Ligprep of Schrödinger suite. Bond orders were refined, missing hydrogen atoms were added followed by generating 3D structures with possible ligand ionization and tautomeric states at pH 7.0 ± 2.0 using

Epik module. The generated low energy conformers were finally energy minimized by using OPLS_2005 force field.

**Protein preparation for *in-silico* docking studies:** The 3D X-ray structure of human GRP78 ATPase domain complexed with 2'-deoxy-ADP and inorganic phosphate (5F0X.*pdb*, Resolution: 1.6 Å) was retrieved from protein data bank and was further prepared using protein preparation wizard of Schrödinger suite 2015-3. The initial protein structure was a homo dimer, where the redundant chains have been removed with deleting waters, refining bond orders and addition of hydrogens. Prime module was used for adding missing side chains and loops followed by generating protonation and tautomeric states of acidic and basic residues at normal pH 7.0 by PROPKA. Next, protein hydrogen bond assignment was done along with side chain flipping of His, Asp and Glu with reorienting hydroxyl and thiol groups. Finally, protein minimization was performed using OPLS_2005 (Optimized Potentials for Liquid Simulations) molecular force field with RMSD of crystallographic heavy atoms kept at 0.30 Å. The quality of prepared protein was validated using Ramachandran plot.

**Grid generation and molecular docking:** A Grid box was generated at the centroid of active site keeping receptor van der Waals scaling of 1.0 with partial charge cutoff at 0.25. The generated low energy conformers were docked into the active site of 5F0X.*pdb* using extra precision mode (XP) docking of Glide (Glide v 6.8, Schrödinger 2015-3) keeping default parameters. The docked pose was selected based on terms of Glide g score, Glide model and Glide energy values.

# Cell viability assay and synergism with tunicamycin

Cell viability was measured by the colorimetric 3-(4,5-dimethyl-2-thiazolyl)-2,5diphenyltetrazolium bromide (MTT) assay. Cells were seeded in 96-well plates at a density of 10^4^ cells per well and treated with vitexin and tunicamycin. 100 μL of 1 mg/mL MTT (Sigma) in

DMEM medium containing 10% fetal bovine serum was added to treated cells for 4 h at 37 °C.

The medium was replaced with 200 μL of DMSO and shaken for 15 min, then absorbance at 540 nm was measured using a microplate ELISA reader with DMSO used as the blank. To quantify the synergistic or antagonist effect of the drugs combinations, Combenefit® software was used.

# In vivo mouse xenograft and vitexin treatment

Four- to six-week-old female balb/c athymic (nuþ/nuþ) mice were purchased from The Charles River Laboratories. The research protocol was approved, and mice were maintained in accordance with the institutional guidelines of the Università degli Studi della Campania L. Vanvitelli Animal

Care and Use Committee. Animal care was in compliance with Italian (Decree 116/92) and European Community (E.C. L358/1 18/12/86) guidelines on the use and protection of laboratory animals. Mice were acclimatized at Università degli Studi della Campania L. Vanvitelli Medical School Animal Facility for 1 week prior to being injected with cancer cells and then caged in groups of 3. A total of 5x 10^6^ MCF-7 cells were resuspended in 200 μL of Matrigel (BD Biosciences) and PBS (1:1) and implanted subcutaneously into the right flank of 12 nude female mice. At week 2, once tumors reached a mean volume of 600 mm^3^, mice were randomized into treatment group (6 mice) or control group (6 mice), to receive treatment with vitexin 2 mg/kg or vehicle (dimethyl sulfoxide (DMSO)), respectively, via intraperitoneal injection, 5 days a week, for 3 weeks. Tumor size was evaluated twice a week by caliper measurements using the following formula: p/6 x larger diameter x (smaller diameter)^2^. Tumor response was assessed by using volume measurements and adapted clinical criteria.

# Statistical Analysis

Statistical analyses were performed using GraphPad Prism Software (Version 8.0). Unpaired Student’s *t* test or two-way anova with Bonferroni Posthoc test was conducted for all the datasets as indicated in figure legends to determine statistical significance. All the data are represented as mean± SEM. For all tests, a *P* value <0.05 was considered statistically significant (* p<0.05; ** p<0.01; *** p<0.001; **** p<0.0001).

# References

1. Semenza GL. The hypoxic tumor microenvironment: A driving force for breast cancer progression. Biochimica et Biophysica Acta (BBA)-Molecular Cell Research. 2016;1863(3):382-91.
2. Joyce JA, Fearon DT. T cell exclusion, immune privilege, and the tumor microenvironment. Science. 2015;348(6230):74-80.
3. Spill F, Reynolds DS, Kamm RD, Zaman MH. Impact of the physical microenvironment on tumor progression and metastasis. Current opinion in biotechnology. 2016;40:41-8.
4. Chipurupalli S, Kannan E, Tergaonkar V, D’Andrea R, Robinson N. Hypoxia induced ER stress response as an adaptive mechanism in cancer. International journal of molecular sciences.

2019;20(3):749.

1. Muz B, de la Puente P, Azab F, Azab AK. The role of hypoxia in cancer progression, angiogenesis, metastasis, and resistance to therapy. Hypoxia. 2015;3:83.
2. Majmundar AJ, Wong WJ, Simon MC. Hypoxia-inducible factors and the response to hypoxic stress. Molecular cell. 2010;40(2):294-309.
3. Graham K, Unger E. Overcoming tumor hypoxia as a barrier to radiotherapy, chemotherapy and immunotherapy in cancer treatment. International journal of nanomedicine. 2018;13:6049.
4. Koritzinsky M, Levitin F, van den Beucken T, Rumantir RA, Harding NJ, Chu KC, et al. Two phases of disulfide bond formation have differing requirements for oxygen. J Cell Biol. 2013;203(4):615-27.
5. Wang M, Kaufman RJ. The impact of the endoplasmic reticulum protein-folding environment on cancer development. Nature Reviews Cancer. 2014;14(9):581-97.
6. Hetz C, Papa FR. The unfolded protein response and cell fate control. Molecular cell. 2018;69(2):169-81.
7. Wang M, Kaufman RJ. Protein misfolding in the endoplasmic reticulum as a conduit to human disease. Nature. 2016;529(7586):326.
8. Walter P, Ron D. The unfolded protein response: from stress pathway to homeostatic regulation. Science. 2011;334(6059):1081-6.
9. Kopp MC, Larburu N, Durairaj V, Adams CJ, Ali MM. UPR proteins IRE1 and PERK switch BiP from chaperone to ER stress sensor. Nature structural & molecular biology. 2019;26(11):1053-62. 14. Lewy TG, Grabowski JM, Bloom ME. Focus: Infectious Diseases: BiP: Master Regulator of the Unfolded Protein Response and Crucial Factor in Flavivirus Biology. The Yale journal of biology and medicine. 2017;90(2):291.
10. White E. The role for autophagy in cancer. The Journal of clinical investigation. 2015;125(1):42-

6.

1. Kroemer G, Mariño G, Levine B. Autophagy and the integrated stress response. Molecular cell. 2010;40(2):280-93.
2. Lin Y, Jiang M, Chen W, Zhao T, Wei Y. Cancer and ER stress: Mutual crosstalk between autophagy, oxidative stress and inflammatory response. Biomedicine & Pharmacotherapy.

2019;118:109249.

1. Anding AL, Baehrecke EH. Cleaning house: selective autophagy of organelles. Developmental cell. 2017;41(1):10-22.
2. White E, DiPaola RS. The double-edged sword of autophagy modulation in cancer. Clinical cancer research. 2009;15(17):5308-16.
3. Li Y-Y, Feun LG, Thongkum A, Tu C-H, Chen S-M, Wangpaichitr M, et al. Autophagic mechanism in anti-cancer immunity: Its pros and cons for cancer therapy. International journal of molecular sciences. 2017;18(6):1297.
4. Hou W, Zhang Q, Yan Z, Chen R, Zeh Iii H, Kang R, et al. Strange attractors: DAMPs and autophagy link tumor cell death and immunity. Cell death & disease. 2013;4(12):e966.
5. Bernales S, McDonald KL, Walter P. Autophagy counterbalances endoplasmic reticulum expansion during the unfolded protein response. PLoS biology. 2006;4(12):e423.
6. Hayashi-Nishino M, Fujita N, Noda T, Yamaguchi A, Yoshimori T, Yamamoto A. A subdomain of the endoplasmic reticulum forms a cradle for autophagosome formation. Nature cell biology.

2009;11(12):1433.

1. Ylä-Anttila P, Vihinen H, Jokitalo E, Eskelinen E-L. 3D tomography reveals connections between the phagophore and endoplasmic reticulum. Autophagy. 2009;5(8):1180-5.
2. Grumati P, Dikic I, Stolz A. ER-phagy at a glance. J Cell Sci. 2018;131(17):jcs217364.
3. Reggio A, Buonomo V, Berkane R, Bhaskara RM, Tellechea M, Peluso I, et al. Role of FAM134 paralogues in endoplasmic reticulum remodeling, ER-phagy, and Collagen quality control. EMBO reports.

2021;22(9). doi: 10.15252/embr.202052289.

1. Khaminets A, Heinrich T, Mari M, Grumati P, Huebner AK, Akutsu M, et al. Regulation of endoplasmic reticulum turnover by selective autophagy. Nature. 2015;522(7556):354-8.
2. Grumati P, Morozzi G, Hölper S, Mari M, Harwardt M-LI, Yan R, et al. Full length RTN3 regulates turnover of tubular endoplasmic reticulum via selective autophagy. elife. 2017;6:e25555.
3. Fumagalli F, Noack J, Bergmann TJ, Cebollero E, Pisoni GB, Fasana E, et al. Translocon component Sec62 acts in endoplasmic reticulum turnover during stress recovery. Nature cell biology.

2016;18(11):1173-84.

1. Smith MD, Harley ME, Kemp AJ, Wills J, Lee M, Arends M, et al. CCPG1 is a non-canonical autophagy cargo receptor essential for ER-phagy and pancreatic ER proteostasis. Developmental cell.

2018;44(2):217-32. e11.

1. Chen Q, Xiao Y, Chai P, Zheng P, Teng J, Chen J. ATL3 is a tubular ER-phagy receptor for GABARAP-mediated selective autophagy. Current Biology. 2019;29(5):846-55. e6.
2. An H, Ordureau A, Paulo JA, Shoemaker CJ, Denic V, Harper JW. TEX264 is an endoplasmic reticulum-resident ATG8-interacting protein critical for ER remodeling during nutrient stress. Molecular cell. 2019;74(5):891-908. e10.
3. Chino H, Hatta T, Natsume T, Mizushima N. Intrinsically disordered protein TEX264 mediates ERphagy. Molecular cell. 2019;74(5):909-21. e6.
4. Delorme-Axford E, Popelka H, Klionsky DJ. TEX264 is a major receptor for mammalian reticulophagy. Taylor & Francis; 2019.
5. Nthiga TM, Kumar Shrestha B, Sjøttem E, Bruun JA, Bowitz Larsen K, Bhujabal Z, et al. CALCOCO 1 acts with VAMP-associated proteins to mediate ER-phagy. The EMBO Journal. 2020:e2019103649.
6. Stephani M, Picchianti L, Gajic A, Beveridge R, Skarwan E, Sanchez De Medina Hernandez V, et al. A cross-kingdom conserved ER-phagy receptor maintains endoplasmic reticulum homeostasis during stress. eLife. 2020;9. doi: 10.7554/elife.58396.
7. Muñoz-Sánchez J, Chánez-Cárdenas ME. The use of cobalt chloride as a chemical hypoxia model. Journal of Applied Toxicology. 2019;39(4):556-70.
8. Tan Q, Wang M, Yu M, Zhang J, Bristow RG, Hill RP, et al. Role of autophagy as a survival mechanism for hypoxic cells in tumors. Neoplasia. 2016;18(6):347-55.
9. Verfaillie T, Salazar M, Velasco G, Agostinis P. Linking ER stress to autophagy: potential implications for cancer therapy. International journal of cell biology. 2010;2010.
10. Bernales S, Schuck S, Walter P. ER-phagy: selective autophagy of the endoplasmic reticulum. Autophagy. 2007;3(3):285-7.
11. Schuck S, Gallagher CM, Walter P. ER-phagy mediates selective degradation of endoplasmic reticulum independently of the core autophagy machinery. J Cell Sci. 2014;127(18):4078-88.
12. Wilkinson S. ER-phagy: shaping up and destressing the endoplasmic reticulum. The FEBS Journal. 2019. doi: 10.1111/febs.14932.
13. Hughes SJ, Antoshchenko T, Chen Y, Lu H, Pizarro JC, Park H-W. Probing the ATP site of GRP78 with nucleotide triphosphate analogs. PloS one. 2016;11(5):e0154862.
14. Grumati P, Dikic I, Stolz A. ER-phagy at a glance. Journal of Cell Science. 2018;131(17):jcs217364.

doi: 10.1242/jcs.217364.

1. Jing X, Yang F, Shao C, Wei K, Xie M, Shen H, et al. Role of hypoxia in cancer therapy by regulating the tumor microenvironment. Molecular cancer. 2019;18(1):157.
2. Wu D, Yotnda P. Induction and testing of hypoxia in cell culture. JoVE (Journal of Visualized Experiments). 2011;(54):e2899.
3. Iurlaro R, Muñoz-Pinedo C. Cell death induced by endoplasmic reticulum stress. The FEBS journal. 2016;283(14):2640-52.
4. Hetz C. The unfolded protein response: controlling cell fate decisions under ER stress and beyond. Nature reviews Molecular cell biology. 2012;13(2):89.
5. Bravo R, Parra V, Gatica D, Rodriguez AE, Torrealba N, Paredes F, et al. Endoplasmic reticulum and the unfolded protein response: dynamics and metabolic integration. International review of cell and molecular biology. 301: Elsevier; 2013. p. 215-90.
6. Pankiv S, Clausen TH, Lamark T, Brech A, Bruun J-A, Outzen H, et al. p62/SQSTM1 binds directly to Atg8/LC3 to facilitate degradation of ubiquitinated protein aggregates by autophagy. Journal of biological chemistry. 2007;282(33):24131-45.
7. Ichimura Y, Kominami E, Tanaka K, Komatsu M. Selective turnover of p62/A170/SQSTM1 by autophagy. Autophagy. 2008;4(8):1063-6.
8. Liu L, Feng D, Chen G, Chen M, Zheng Q, Song P, et al. Mitochondrial outer-membrane protein

FUNDC1 mediates hypoxia-induced mitophagy in mammalian cells. Nature cell biology. 2012;14(2):177.

1. Walter KM, Schönenberger MJ, Trötzmüller M, Horn M, Elsässer H-P, Moser AB, et al. Hif-2α promotes degradation of mammalian peroxisomes by selective autophagy. Cell metabolism.

2014;20(5):882-97.

1. Dikic I. Open questions: why should we care about ER-phagy and ER remodelling? BMC biology. 2018;16(1):131.
2. Loi M, Fregno I, Guerra C, Molinari M. Eat it right: ER-phagy and recovER-phagy. Biochemical Society Transactions. 2018;46(3):699-706.
3. Ng KP, Manjeri A, Lee KL, Huang W, Tan SY, Chuah CT, et al. Physiologic hypoxia promotes maintenance of CML stem cells despite effective BCR-ABL1 inhibition. Blood. 2014;123(21):3316-26.
4. Daskalaki I, Gkikas I, Tavernarakis N. Hypoxia and selective autophagy in cancer development and therapy. Frontiers in cell and developmental biology. 2018;6.
5. Otero JH, Lizák B, Hendershot LM, editors. Life and death of a BiP substrate. Seminars in cell & developmental biology; 2010: Elsevier.
6. Srivastava R, Deng Y, Shah S, Rao AG, Howell SH. BINDING PROTEIN is a master regulator of the endoplasmic reticulum stress sensor/transducer bZIP28 in Arabidopsis. The Plant Cell. 2013;25(4):141629.
7. Forrester A, De Leonibus C, Grumati P, Fasana E, Piemontese M, Staiano L, et al. A selective ERphagy exerts procollagen quality control via a Calnexin-FAM134B complex. The EMBO journal.

2019;38(2).

1. Hübner CA, Dikic I. ER-phagy and human diseases. Cell Death & Differentiation. 2019:1-10. 62. Xiao J, Capanoglu E, Jassbi AR, Miron A. Advance on the flavonoid C-glycosides and health benefits. Critical reviews in food science and nutrition. 2016;56(sup1):S29-S45.
2. Hassan WH, Abdelaziz S, Al Yousef HM. Chemical Composition and Biological Activities of the Aqueous Fraction of Parkinsonea aculeata L. Growing in Saudi Arabia. Arabian journal of chemistry.

2019;12(3):377-87.

1. He M, Min J-W, Kong W-L, He X-H, Li J-X, Peng B-W. A review on the pharmacological effects of vitexin and isovitexin. Fitoterapia. 2016;115:74-85.
2. Itakura E, Mizushima N. Characterization of autophagosome formation site by a hierarchical analysis of mammalian Atg proteins. Autophagy. 2010;6(6):764-76.

# Acknowledgement

We thank the Robinson laboratory members and Dr Alexandra Stolz, Institute of Biochemistry 2, Goethe University School of Medicine, Frankfurt, Germany for helpful discussions and critical review of the manuscript; Dr Makoto Kamei, Imaging Facility, Centre for Cancer Biology,

Adelaide. The imaging facility is supported by funding from Australian Cancer Research Foundation. Dr Chia Chi, Centre for Cancer Biology for helping us to set up hypoxic conditions and Dr Srikanth Jupudi, JSS College of Pharmacy for his help with the In-silico docking studies.

Work in the laboratory of NR is supported by funds from Centre for Cancer Biology and University of South Australia and from Cologne Excellence Cluster on Cellular Stress Responses in AgingAssociated Diseases (CECAD; funded by the DFG within the Excellence Initiative by the German federal and state governments) and Köln Fortune, University clinic, Cologne, grants from

Deutsche Forschungsgemeinschaft (SFB 670). We thank Dr. Elena Polishchuk and the Microscopy

Core of TIGEM for electron microscopy analysis. We thank Dr. Luca Persano and Dr. Elena Mariotto for their help in hypoxia treatments. The authors acknowledge Department of Science and Technology, Government of India for financial support vide reference no SR/WOS-A/LS21/2016 under Women Scientist Scheme to carry out research in this area to SC. SC was also supported with a short-term travel fellowship from European Association for Cancer Research. The authors acknowledge Telethon Foundation and AIRC (MFAG 24856). AR was supported by Fondazione Umberto Veronesi.

**Competing Financial interest Statement**

The authors declare no Competing Financial interests.

# Figure Legends

**Fig. 1: Hypoxia induces ER stress response and autophagy (a)** Immunoblot showing stabilization of HIF-1a during CI-hypoxia (500uM CoCl2 for 24h) *(n=3)*. **(b)** Proliferation of

MCF-7 cells upon CI-hypoxia and (c) HE compared to normoxia expressed as fold change *(n=5)*.

**(d)** Confocal microscopy image of MCF-7 cells in normoxia and HE transfected with mCherryER-3 plasmid (Cyan). **(e)** Transmission Electron Microscopy (TEM) image of stressed annulated ER upon CI- Hyp and **(f)** HE. **(g)** Expression of UPR proteins upon CI-hypoxia and **(h)** cells grown in hypoxic environment (HE) (1% O_2_) compared to normoxia *(n=3)*.

# Fig. 2: Hypoxia induces ER-phagy to maintain ER homeostasis

**(a)** Immunoblot of LC3I to LC3II conversion and p62 upon concanamycin A treatment during CIhypoxia in comparison with control *(n=3).* **(b)** MCF-7 cells subjected to CI-hypoxia and stained for calnexin (ER) and WIPI (autophagosomes). **(c)** MMTV-pyMT mouse breast cancer tissue section stained for LC3B (green) and HIF-1a (magenta). (d) Immunoblot of p62 upon CI-hypoxia and **(e)** cells grown in HE (1% O_2_). **(f)**TEM image of CI and HE hypoxic cells treated with concanamycin A to prevent autoloysosomal degradation. White arrows and blac arrows denote autolysosomes and ER fragments in autolysosomes respectively. **(g)** Immunoblot of FAM134B during CI-hypoxia and HE (1% O_2_) compared to normoxia *(n=5)*. **(h)** Western blot analysis of FAM134B expression in the presence of CoCl_2_ and after CoCl_2_ wash out *(n=3)*. **(i)** Western blot of FAM134B during HE (1% O2) in the presence and absence of concanamycin A *(n=4)*. **(j)** Doxycycline induced HA-tagged FAM134B inMCF-7 cells were subjected with CI-hypoxia or

HE in the presence and absence of concanamycin A were stained for HA (green) and LC3B (magenta), nucleus was stained with DAPI (blue) and imaged using a confocal microscope. **(k)**

Confocal immunofluorescence image of MMTV-pyMT breast cancer tissue section stained for

FAM134B (red), LC3B (green) and nucleus (blue). **(l)** Co-immunoprecipitation of LC3B with

FAM134B during hypoxia compared to normoxia *(n=3)*. **(m)** Immunoblot of UPR proteins upon *FAM134B* knockdown using specific siRNAs *(n=3)*. **(n)** Relative % cell viability of hypoxic cells compared to normoxic cells depleted of FAM134B using specific siRNA *(n=5)*.

**Fig. 3: Hypoxia induced ER-phagy is BiP-dependent (a)** Immunoblot of UPR proteins in MCF7 cells transfected with HIF-1a siRNA or control siRNA and subjected to CI-hypoxia *(n=3).* **(b-e)** Densitometric quantification of UPR proteins; **(b)** BiP **(c)** XBP-1s **(d)** ATF4 **(e)** CHOP in MCF-7 cells transfected with HIF-1a siRNA or control siRNA and subjected to CI-hypoxia *(n=3)*. **(f)** Western blot analysis of FAM134B in HIF-1a-depleted cells during CI-hypoxia and **(g)** HE (1%

O2) *(n=3)*. **(h)** Western blot analysis of FAM134B in BiP knock down cells using siRNA during CI-hypoxia *(n=3)*. **(i)** Confocal immunofluorescence image of MCF-7 cells cultured in HE stained for FAM134B (green), BiP (purple) and nucleus – DAPI (blue); scale bar = 10 µm. **(j)** Confocal microscopy of MMTV-pyMT breast cancer tissue section stained for FAM134B and BiP; scale bar = 20um. **(k)** Confocal microscopy of human breast cancer tissue section stained for FAM134B and BiP; scale bar = 100um. **(l)** Immunoblot illustrating co-immunoprecipitation of BiP with FAM134B during hypoxia compared to normoxia *(n=3)*. **(m)**  Crystal violet cell proliferation assay (absorbance is shown) on MCF-7 cells in HE compared to normoxia *(n=6).*

**Fig 4: *In-Silico* molecular modeling and dynamic simulation studies identified vitexin as a potential BiP inhibitor (a)** Putative binding mode of the vitexin in the crystal structure of BiP. **(b)** The important residues in the binding pocket and **(c)** depicting the residues that may be important for the interaction with vitexin. Carbon atoms of vitexin are colored green and the important residues in the binding pocket are colored in gray. Oxygen atoms are colored in red, nitrogen atoms in blue, and sulfur atoms in yellow. **(d)** Root mean square deviation (RMSD) values of BiP (blue) and in complex with vitexin (red) over 100 ns. The values were obtained from the Cα atoms relative to the conformation of the first frame. **(e)** Root mean square fluctuation (RMSF) values of BiP (blue) and in complex with vitexin (red) over 100ns.

# Fig 5: Vitexin prevents FAM134B-BiP interaction and inhibits ER-phagy (a) Immunoblot of

BiP upon vitexin (20µM) treatment during CI-hypoxia when compared to normoxia. **(b)** Densitometric quantification of BiP upon vitexin (20µM) treatment during CI-hypoxia when compared to normoxia. **(c)** Coimmunoprecipitation of FAM134B with BiP upon vitexin treatment during CI-hypoxia *(n=2)*. **(d)** Confocal immunofluorescence image of MCF-7 cells treated with vitexin cultured in HE stained for FAM134B (green), BiP (purple) and nucleus – DAPI (blue); scale bar = 10 µm. (e) Immunoblot of FAM134B upon vitexin treatment during CI-hypoxia. **(f)** Densitometric quantification of FAM134B upon vitexin treatment during CI-hypoxia. (g) Immunoblot of FAM134B in MCF7 cells culture in HE (1% O_2_) upon vitexin treatment and (h) densitometric quantification.

**Fig 6: Vitexin reduces tumor burden in breast cancer xenograft mouse model (a)** Crystal violet cell viability assessment upon vitexin treatment during CI-hypoxia *(n=3)*. **(b)**Synergistic effect of vitexin with tunicamycin analyzed using Combenefit® software**. (c)** Representative images of tumor grafts from female balb/c athymic (nuþ/nuþ) mice 21 days following an injection of vitexin and control *(n=6)*. **(d)** Tumor volumes in control and vitexin treated xenograft mice

748 recorded on the days shown in the graph *(n=6)*. **(e)** Representation of FAM134B-BiP complex 749 mediated ER-phagy activated upon hypoxia induced accumulation of misfolded/unfolded proteins.

750
